# Consumer Preference Segments for Plant-Based Foods: The Role of Product Category

**DOI:** 10.3390/foods11193059

**Published:** 2022-10-01

**Authors:** Armand V. Cardello, Fabien Llobell, Davide Giacalone, Sok L. Chheang, Sara R. Jaeger

**Affiliations:** 1 A.V. Cardello Consulting and Editing Services, Framingham, MA 01701, USA; 2 Addinsoft, XLSTAT, 75018 Paris, France; 3 SDU Innovation & Design Engineering, Department of Technology and Innovation, University of Southern Denmark, 5230 Odense, Denmark; 4 The New Zealand Institute for Plant and Food Research Limited, Mt Albert Research Centre, Private Bag 92169, Auckland 1142, New Zealand

**Keywords:** plant-based foods, consumer research, preference segments, product category, milk, cheese, fish, meat

## Abstract

A survey of willingness to consume (WTC) 5 types of plant-based (PB) food was conducted in USA, Australia, Singapore and India (*n* = 2494). In addition to WTC, emotional, conceptual and situational use characterizations were obtained. Results showed a number of distinct clusters of consumers with different patterns of WTC for PB foods within different food categories. A large group of consumers did not discriminate among PB foods across the various food categories. Six smaller, but distinct clusters of consumers had specific patterns of WTC across the examined food categories. In general, *PB Milk* and, to a much lesser extent, *PB Cheese* had highest WTC ratings. *PB Fish* had the lowest WTC, and two PB meat products had intermediate WTC. Emotional, conceptual and situational use characterizations exerted significant lifts/penalties on WTC. No penalty or lifts were imparted on WTC by the situational use of ‘moving my diet in a sustainable direction’, whereas uses related to ‘when I want something I like’ and ‘when I want something healthy’ generally imparted WTC lifts across clusters and food categories. The importance of this research for the study of PB foods is its demonstration that consumers are not monolithic in their willingness to consume these foods and that WTC is often a function of the food category of the PB food.

## 1. Introduction

Over the past decades, it has become increasingly clear that the consumption of animal products has had unsustainable effects on the environment through high demand on land, water, feed, housing and the production of greenhouse gases [1,2,3,4,5,6,7,8,9,10]. In addition, excess consumption of animal products is known to have harmful effects on human health, including cancer, cardiovascular disease and obesity [11,12,13,14,15]. In response to these known detrimental effects of animal protein consumption on the environment and human health, global food policy has shifted to place greater emphasis on more sustainable farming practices and protein sources [1,16,17]. Leading this trend has been the emphasis on plant-based protein consumption [18,19,20]. This trend has resulted in a dramatic increase in the number of plant-based foods, beverages, product extenders and/or meat alternatives now available globally. It is estimated that the plant-based food market will further grow from approximately $US 30 billion in 2020 to $US 160 billion by 2030 [21]. In addition to plant-based proteins, a variety of other alternative proteins are now being investigated for their potential to replace animal protein in foods, e.g., algae, insects, and cultured meat [22,23,24,25,26,27,28].

### 1.1. Factors Influencing Acceptance of Plant-Based and Other Alternative Proteins

With the rapid growth in the alternative protein food market, research into the factors that drive consumer choice, purchase, acceptance and consumption of foods containing these proteins has grown correspondingly. Among the many factors that influence consumer acceptance and choice behavior toward these products are their sensory properties, e.g., taste, odor, texture, appearance [29,30,31,32,33,34,35,36,37,38], their familiarity [23,25,26,39,40,41,42,43,44], price/brand [37,45,46,47] and how appropriate the alternative protein is within the meal or its context of use [37,48,49,50,51].

Other important factors relate more specifically to the individual. These include the health concerns of individuals and the perceived benefits attributed to the alternative protein [52,53,54,55,56,57,58,59,60], the consumers’ attitudes [36,44,55,61,62], their values and cultural norms [3,63,64,65,66,67,68] and their neophobic tendencies [25,28,32,38,40,44,69,70,71,72,73].

Due to the important impact of individual factors in alternative protein acceptance, several studies have searched for distinct segments of consumers with different preferences for alternative proteins. Thus, de Boer et al. [29] examined consumer involvement with food and its influence on the acceptance of alternative proteins. These researchers identified ‘trendsetters’ who sought more authentic proteins, like lentils and seaweeds, but eschewed hybrid products like meat extended with plant proteins [29]. Van der Zanden et al. [74] examined preference segments among elderly consumers and found that the type of carrier (food type) for the protein was an important differentiator among different clusters of consumers, as was the meal type (meal component vs. snack). Health-orientation was another important variable differentiating a majority of consumer segments in a study of seaweed protein consumption by Palmieri and Forleo [75], as well as in a study by Possidónio et al. [76] who identified 3 segments of alternative protein consumers; (1) hedonically motivated meat eaters uninterested in meat substitutes, (2) health-oriented meat eaters open to some meat substitutes, and (3) ethically conscious meat avoiders positive toward protein alternatives to meat. Finally, in a study by Aschemann-Witzel and Peschel [34], individuals who previously purchased or consumed vegetarian products had more differentiated associations to different protein types, suggesting that different segments of consumers may respond differentially to plant proteins depending on the category of food.

Despite the environmental and health reasons that support consumption of alternative protein foods, research has shown that consumer acceptance and choice behavior toward these products is lower than for their animal counterparts [77,78,79,80]. Despite the need to identify alternative proteins with the greatest potential for acceptance by consumers, most research has examined consumer attitudes and acceptance within the context of a single protein source, e.g., plants, pulses, algae, seaweed, insects, etc., and relatively fewer have studied multiple alternative proteins [80]. Among studies comparing multiple proteins, results have generally shown that plant-based proteins are more acceptable to consumers than other protein sources, e.g., seaweed and cultured meat, and that insect protein is the least preferred [47,53,76,81,82,83,84]. Nevertheless, a recent review of the literature on alternative proteins found that the vast majority of published research studies focused on insect protein and far fewer examined plant proteins [80].

### 1.2. Role of Food Product and Food Category 

Although several studies have examined different alternative proteins or protein mixes within the same product, e.g., hamburger, milk alternatives, lasagna [47,48,82,85,86], relatively few have examined differences in acceptance/willingness to consume as a function of product type, in spite of the fact that in their review of the area, Hartmann and Siegrist [77] found that acceptance of alternative proteins varies by product and that “it is of little value to ask consumers about abstract concepts (e.g., are you willing to eat insects?”). Still fewer studies have compared consumer reactions to alternative proteins based on differences in the target food group or category (e.g., milk, cheese, meat, fish, etc.). This, too, in spite of the fact that the food category to which a product belongs has been shown to have a strong influence on consumer responses to alternative proteins.

In one series of studies, Elzerman et al. [49,50,87] showed that alternative proteins differ in acceptance depending upon the food (or food category) in which they are incorporated. For example, consumers were found to have a greater willingness to accept meat substitutes when served with spaghetti than as part of a soup. Similarly, Michel et al. [37] observed significant interactions between protein type (meat vs. non-meat) and product groups in the cognitive associations to different test products (e.g., nuggets, sausages). In still another study on alternative plant proteins, Aschemann-Witzel and Peschel [34] found differences in consumer perceptions of these products depending on food category. In their study, consumers were shown ingredient lists that accompanied sketches of two different products identified as being in the food categories of “protein drink” or “sorbets.” The ingredient list for each product category was varied to include either the term ‘protein’, ‘vegetable protein’, ‘soy protein’, ‘pea protein’ or ‘potato protein’. Results showed that the product category evoked specific associations for the protein(s), e.g., ‘nutrition’ for protein drinks and ‘functional’ for sorbets, and that more positive associations accrued to the protein drinks and more negative associations to the sorbets. Further, the nature of the protein evoked different associations depending on the food category in which it appeared. Functional roles, e.g., serving as a cohesive ingredient, were evoked for the potato and pea proteins, but only within the sorbet category. 

The food category to which a specific food belongs is an important factor influencing consumer behavior toward it, because it serves a variety of purposes and needs for the consumer [34]. Primary among these is that food categories establish the context within which the product is conceptualized by the consumer. For example, ‘salmon’, as a product, is conceptually a member of the food category ‘fish’ and, thereby, accrues a variety of cognitive, emotional and situational use associations and expectations that are shared by all types of fish, e.g., specific sensory (odor, texture) expectations, expectations related to preparation, cooking, etc. Similarly, for insect proteins, although, ants, beetles, and mealworms all have different sensory attributes, consumers react to these products in a similar way, simply because they are all members of the ‘insect food’ category, which evokes both generalized neophobic and disgust responses in many individuals [88,89,90,91,92,93]. Even in routine CLT tests on familiar products, it has been shown that over 75% of consumers, when asked after the test whether they rated their liking of that food within the context of ‘this kind of food’ or ‘all foods’, reported that their ratings were made within the context of ‘this *kind* of food’ [48]. 

To our knowledge, it has not previously been shown across a range of possible food categories that consumers hold distinct PB food category preferences or that (for example) a high WTC for one PB food category is an unreliable predictor of WTC (and preferences) for other PB food categories. However, if true, this finding would have implications for the promotion of PB diets, since it could imply that promotional campaigns, if category generic, may resonate with fewer people than a more category-targeted campaign. Thus, one goal of the present research is to explore the degree to which WTC for PB foods is dependent on the food category to which the PB food belongs. 

### 1.3. Objectives and Overview of the Empirical Approach

Building on the above research threads, the specific objectives of the present research were: (1) to examine consumers’ willingness to consume plant-based protein within a number of distinct food categories, (2) to determine if there are different segments of consumers who have different patterns of their willingness to consume plant-based proteins, and to (3) assess differences in the identified segments of consumers in terms of their emotional, cognitive or situational use characterizations of the different plant-based categories of food and the impact of these variables on their judgments of willingness to consume these products. The latter objective is aimed at filling the literature gap identified by Onwezen et al. [94] in which it was noted that, with minor exceptions [37,82,94] few studies have examined either the role of emotions or other affective variables on acceptance of alternative proteins or the physical or social environment in which consuming such proteins is most appropriate or contextually acceptable.

## 2. Materials and Methods

### 2.1. Participants 

Consumer insights for a global challenge like sustainable food production are more comprehensive when research is conducted in multiple countries. For this reason, participants in the present research came from the United States of America (US, *n* = 629), the Commonwealth of Australia (AU, *n* = 623), the Republic of Singapore (SG, *n* = 627), and the Republic of India (IN, *n* = 615). These countries differed on several dimensions including geographical location, population size, national cultures [95], importance of F&B sectors in national economies [96], sustainable energy [97], proportion of people following a vegetarian/vegan diet [98,99,100,101], growth rates of plant-based foods in retail [102], as well as the regulatory and legal policies regarding the labeling of PB foods. Country selection was also informed by a desire to lessen the dominance of Sensory-Consumer research taking place in Western, Educated, Industrialized, Rich, and Democratic (WEIRD) and especially Anglocentric countries [103].

Participants had self-registered on a database managed by a web panel provider with ISO 20252:2019 accreditation (ISO: International Organization for Standardization [104]). A quota sampling strategy was imposed by country with interlocking quota for men (50%) and women (50%) across two age groups (18–45 y.o. (50%), 46–69 y.o. (50%)). The samples were not nationally representative, but diverse across a range of characteristics such as living location, educational attainment, marital status, etc. (Table 1) (Part S1 of Supplementary Materials has country specific details). High proficiency in English and regular participation household grocery shopping and food preparation (more than once a week) were imposed as eligibility criteria. 

#### Human Ethics Statement

The study was covered by a general approval for sensory and consumer research from the Human Ethics Committee at the New Zealand Institute for Plant and Food Research Limited. Participants gave voluntary consent and were assured that their responses would remain confidential. They were informed that they could end their participation at any time. As compensation, participants received reward points which could be redeemed for online purchases. 

### 2.2. Plant-Based Food Stimuli

The research focused on 5 plant-based (PB) foods (abbreviations for figures and tables in brackets):

- Milk—from 100% plant-based ingredients (*PB Milk*)
- Cheese—from 100% plant-based ingredients (*PB Cheese*)
- Meat—blend containing 33% plant-based ingredients (*PB Meat 33%)*
- Meat—from 100% plant-based ingredients (*PB Meat*)
- Fish—from 100% plant-based ingredients (*PB Fish*)

Milk and cheese were chosen because they are both familiar but quite different sub-categories within the overall dairy category and have quite different levels of availability and consumer acceptance (milk—readily available, well accepted; cheese—less readily available, lower acceptance). Meat (100% and 33% plant-based) were chosen because meat protein is the most highly targeted protein for replacement, but percentage replacement can influence acceptance and willingness to consume. Meat hybrids like *PB Meat 33%* offer a more sustainable food alternative for consumers who consider meat to be an essential and integral element of their daily diet [105]. Lastly, fish was chosen as it is a more novel food category for plant-based products and identified as a niche for PB food innovations by Alcorta et al. [106]. 

To provide a common frame of reference, participants read a short text (written by authors SRJ and DG) before answering questions about the focal foods. It read: “Our current approaches to food production and our consumption levels are global problems because they drive climate change and environmental degradation. Animal farming is particularly damaging for the environment, and to reduce environmental impacts we must change the way we eat—increasing our consumption of plant foods while substantially limiting our intake from animal sources. To support this transition, interest has centered on those plant foods that are good sources of proteins—soybeans, nuts, peas, and some grains—and can provide a nutritionally sound alternative to foods from animals, while also being suitable for the growing number of people who do not, or cannot, eat certain animal-sourced foods (e.g., vegetarians/vegans, people who have a dairy allergy or are lactose intolerant). In the past decade, more and more of these 100% plant-based foods have become available in the [United States/Australia/Singapore/India]. PB “milks” made from, for example, soy, almonds, oats, and even cashew nuts are fairly familiar now, and plant-based “yoghurt” and “cheese” are no longer uncommon. PB “meat”, “fish”, and “seafood” are much more novel. Besides replacing foods from animals with plant-based foods, or creating blends of animal-plant foods, there is also considerable interest in developing alternative sources of animal-derived proteins. These include insects.” 

The text continued to describe other novel types of foods/food technologies (Part S2 of Supplementary Materials has the text in full). The reason was to provide background information for other foods/food technologies also included in the survey but not relevant for the present research (Part S2 of Supplementary Materials lists these other foods).

### 2.3. Empirical Procedures

#### 2.3.1. Stimulus Evaluation

Stimulus evaluation proceeded in two parts. First, the plant-based food names were assessed using two check-all-that-apply (CATA) question [107], pertaining to (i) emotional and cognitive product perceptions, and (ii) situational use product perceptions. This evaluation proceeded sequentially and both CATA questions were completed before the next food name appeared. Randomization of stimuli and terms were used within CATA questions and across participants.

Drawing on a general vocabulary [108], the CATA question relating to emotional and cognitive product conceptualizations included 16 terms: ‘adventurous’, ‘boring’, ‘classy’, ‘comforting’, ‘dissatisfied’, ‘easygoing’, ‘energetic’, ‘enthusiastic’, ‘feminine’, ‘happy’, ‘inspiring’, ‘nervous’, ‘passive’, ‘powerful’, ‘pretentious’, ‘sophisticated’, ‘tense’, ‘uninspired’, ‘unique’, and ‘youthful’. The CATA question relating to situational use included 8 terms: ‘When I want something I like’, ‘When I feel like trying something new’, ‘To move my diet in a more sustainable direction’, ‘When I want something healthy’, ‘As part of meals that I post on social media’, ‘To set a good example to those around me’, ‘As a regular part of my diet’, and ‘As part of easy and convenient meals’. These were developed to address aspects of pleasure, health, environmental concern, social status, fit to diet and convenience, and drew in part on extant literature [109]. The suitability of CATA questions for measuring perceived situational appropriateness was previously demonstrated, for example, by Jaeger et al. [110]. When answering each of these CATA questions, participants were instructed to “Please think about this [stimulus name]. Select all the words that apply.”

The second part of the stimulus evaluation obtained stated willingness to consume, which was measured using the question: “How often would you consume the following foods and beverages?” and a fully labelled 9-pt category scale with anchors: 1 = ‘Never or less than once yearly’, 2 = ‘2–3 times a year’, 3 = ‘Every 2–3 months’, 4 = ‘Once every month’, 5 = ‘1–3 times per month’, 6 = ‘Once every week’, 7 = ‘2–4 times per week’, 8 = ‘5–6 times per week’, and 9 = ‘Once daily or more often’ [109,111]. Stimulus presentation order was randomized.

#### 2.3.2. Psychographic Variables and Dietary Habit

Responses to psychographic variables were obtained after stimulus evaluation. The scales included in all countries were Food Neophobia (FN) [112], Food Technology Neophobia (FTN) [113,114] and environmental concern (ENV) [115]. The 10-item food neophobia scale is widely used to capture consumers’ stable propensity to avoid novel and unfamiliar foods (e.g., ‘I don’t trust new foods’). Across 13 items, FTN measured consumers’ fears of novel food technologies (e.g., ‘new food technologies are something I am uncertain about’ and ‘it can be risky to switch to new food technologies too quickly’). Based on a recent review of scales to measure concern for the environment, a composite scale was created combining four items from each of three existing scales [116,117,118] seeking to mitigate criticisms of existing scales. The constructed scale included 12 items (e.g., ‘If things continue on their present course, we will soon experience a major ecological catastrophe’, ‘I am worried about future children’s chance of living in a clean environment’, and ‘We shouldn’t worry about environmental problems because science and technology will solve them before very long), which are given in full in Part S3 of Supplementary Materials. 

All responses were obtained on fully labelled 7-point Likert scales with anchors: ‘Disagree strongly’ (1), ‘Disagree moderately’ (2), ‘Disagree slightly’ (3), ‘Neither agree nor disagree’ (4), ‘Agree slightly’ (5), ‘Agree moderately’ (6), and ‘Agree strongly’ (7). Participants were instructed to indicate their degree of agreement or disagreement with each of the statements. Within the psychographic scales, statement order was randomized across participants.

Dietary habit was categorized using a question from De Backer and Hudders [119] with nine available options. Participants were classified as Omnivores (no limitation on consumption of meat and fish), Flexitarians (consciously limits quantity of either all types or specific types of meat) or Vegetarians (who completely avoid the consumption of meat and fish).

#### 2.3.3. Data Collection

The survey was conducted in English and was appropriate given the status of this language as *lingua franca* in all four countries. High proficiency in English as an eligibility criterion further ensured that participants had the necessary language skills to complete the survey.

Demographic and socio-economic information was obtained either at the start of the survey (for quota sampling purposes) or at the end of the survey.

Data collection took place in December 2021 and January 2022, following careful revision of test links and evaluation of responses from ~10% of the total sample in each country to ensure that the survey performed as expected. 

The data were obtained as part of a survey that also included other questions, which are not described further due to lack of relevance for the present research. Participants completed the task from a location of their own choosing, using a desktop or laptop computer. 

Drawing on Jaeger and Cardello [120] who identify factors affecting data quality in online questionnaires, data-driven exclusion criteria were implemented relating to completion time and straight-line responding (see Part S4 of Supplementary Materials for details).

### 2.4. Data Analysis

All analyses were performed in XLSTAT [121], using a 5% significance level for inference tests.

#### 2.4.1. Willingness to Consume

As directed by Obj. 1 and the exploration of consumers’ WTC for PB foods, an ANOVA followed by Tukey’s HSD multiple comparisons was performed with WTC ratings as the dependent variable and PB food category as the explanatory variable. In a second step, violin plots [122] were drawn to show the heterogeneity of the distributions of WTC ratings between PB food categories. 

#### 2.4.2. Emotional, Conceptual and Situational Use Terms

Extending the analyses linked to Obj. 1, consumer-derived profiling of the PB food categories was explored. Upon confirming that each CATA term was discriminant via Cochran’s Q tests, Correspondence Analysis was applied on the PB category x term contingency tables [123]. 

To determine the effect of each term on the WTC ratings, penalty/lift analysis was performed [123]. The purpose is to determine for each of the PB food categories which terms positively or negatively affect WTC. The change in WTC for each PB food is calculated and a student’s test between the average WTC when the term is checked and when the term is unchecked to establish if this difference is significantly different from zero.

#### 2.4.3. Consumer Segmentation

To perform consumer segmentation (Obj. 2), an agglomerative hierarchical clustering based on the WTC scores across the 5 PB food categories was used. This cluster analysis was computed with the Euclidean distance and Ward’s criterion [124]. To build clusters that discriminated between the PB food categories, a centering of the WTC scores by subject was carried out beforehand. The number of clusters to retain was determined by visual inspection of the dendrogram, where a significant change of within-cluster variation highlighted a merge of two heterogeneous clusters. 

To visualize differences in the WTC patterns between the clusters, the matrix of clusters x PB foods centers of gravity was calculated and submitted to a PCA based on the covariance matrix. Cluster confidence ellipses (95%) were computed by bootstrapping [125]. 

An ANOVA measuring the PB food category effect on the WTC scores was performed for each cluster separately. As the sample size differed greatly between clusters, effect sizes (η^2^) were used in addition to *p*-values as indicators of degree of product discrimination within each cluster. ANOVAs to measure the cluster effect on each of the PB food categories separately, as well as on the category means were also computed and followed by Tukey’s multiple comparisons.

Per Obj. 3, the final step was to perform penalty/lift analysis within each of the retained clusters to determine the impact on average WTC scores on CATA term selection for each of the 5 PB food categories. Within clusters, the analysis was performed in the same manner as described in Section 2.4.2.

#### 2.4.4. Psychographic and Socio-Demographic Variables

For trait scales (FN, FTN) and ENV concern, summed scores for each participant were calculated across all scale items (following reverse coding as needed). Cronbach’s alpha values exceeded the 0.7 threshold for internal reliability [126]. 

To investigate whether the clusters were influenced by psychographic and/or socio-demographic characteristics, an analysis of the proportions of presence of different variables was performed: country, age, gender, education, and dietary preferences.

## 3. Results

### 3.1. Aggregate Level Findings

#### 3.1.1. Willingness to Consume

**Figure 1:**
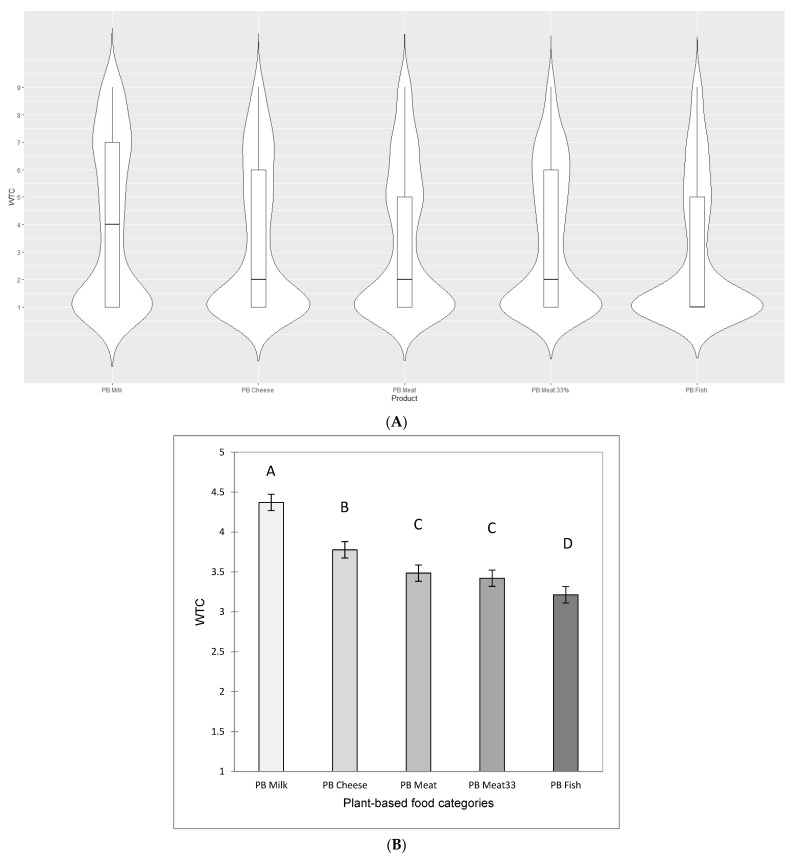
Mean willingness to consume (WTC) ratings for aggregate sample across 2494 consumers in USA, Australia, Singapore and India shown for the five plant-based (PB) food categories included in the research. (**A**) Violin plot of WTC ratings for PB food categories, with median and interquartile range; (**B**) Mean WTC ratings, with standard error. WTC was measured on a 9-point scale (1 = ’Never or less than once yearly’, 5 = ‘1–3 times per month’, 9 = ‘Once daily or more’). In (**B**), PB food categories with different letters following Tukey’s post hoc test are significantly different at the 5% level of significance.

The first objective of the present research was to examine consumers’ willingness to consume plant-based protein within a number of distinct food categories (Obj. 1). Across all countries and PB food categories, slightly more than one-third of all willingness to consume (WTC) ratings (37.3%) were for the response option ‘never or less than once yearly’ (see also Figure 1A), while 9.6% were for the two response options indicating willingness to consume most frequently (‘5–6 times per week’ and ‘once daily or more often’). Figure 1B shows the mean WTC ratings by PB food category for all participants across countries. The *PB Milk* category had the highest WTC (between ‘once every month’ and ‘1–3 times per month’) followed, in order, by *PB Cheese*, *PB Meat* and *PB Meat 33%*. The food category for which there was, on average, the lowest WTC was *PB Fish* (all between ‘every 2–3 months’ and ‘once every month’). Part S5 of Supplementary Materials shows that similar preference ordering was obtained when summarizing and analyzing the data non-parametrically. 

Heterogeneity in WTC ratings for PB food categories was revealed in Figure 1A which shows violin plots for each of the PB food categories. It was particularly obvious that WTC ratings with notably higher than the mean and median values were provided by a modest proportion of participants. This pointed to heterogeneity in consumers’ WTC ratings and paved the way for the second objective of the present research (Obj. 2).

#### 3.1.2. Emotional and Conceptual Product Associations

**Figure 2:**
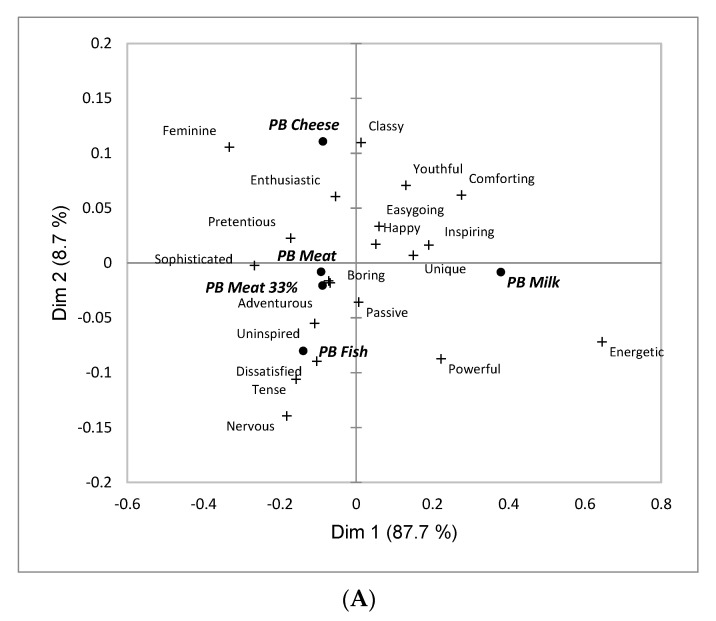

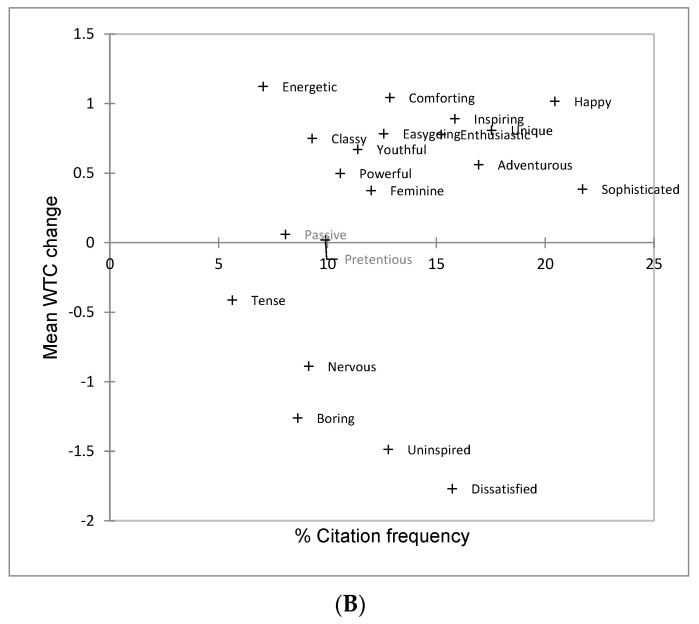
Results linked to emotional and conceptual product associations for aggregate sample across 2494 consumers in USA, Australia, Singapore and India shown for aggregate sample the five plant-based (PB) food categories included in the research. (**A**) Plot of the first two dimensions following Correspondence Analysis; (**B**) Impact on average willingness to consume (WTC) based on Penalty/Lift analysis. In (**A**), PB food categories are shown in bold and italic font. In (**B**), grey font used for terms where change in WTC is not significantly different from zero at the 5% level of significance.

According to Cochran’s Q tests, the five PB food categories were significantly differentiated (*p* < 0.05) on all emotional and conceptual terms except ‘passive’ (*p* = 0.21). Overall, the most frequently used terms were ‘sophisticated’ (22%) and ‘happy’ (20%), while ‘tense’ (6%) and ’energetic’ (7%) were least frequently used (Part S6 of Supplementary Materials has full details). Figure 2A shows a biplot of the two first dimensions after CA, with a dominating first dimension (87.7%) (Part S7 of Supplementary Materials shows the average stimulus positions with 95% confidence intervals). *PB Milk* was separated from the other PB food categories on the first dimension and most strongly associated with ‘energetic’ and ‘powerful’, and least strongly associated with ‘sophisticated’. The second dimension (8.7%) separated *PB Cheese* from *PB Fish*, with the former being significantly more frequently associated with ‘feminine’ and ‘classy’, while negative emotions – ‘dissatisfied’, ‘tense’ and ‘nervous’ were dominant for *PB Fish*. The associations for *PM Meat* and *PM Meat 33%* were very similar.

Penalty/Lift analysis was used to identify the relationships between stimulus characterization and WTC, with Figure 2B showing that selection of ‘happy, ‘comforting’ and ‘energetic’ was associated with a positive change in WTC of more than one scale point, and that selection of ‘boring’, ‘uninspired’ and ‘dissatisfied’ was associated with a negative change in WTC of more than one scale point. Smaller positive WTC changes (0.5 to 1 scale point) were observed for many positive and conceptual terms, while negative WTC change of a similar magnitude was associated with ‘nervous’.

#### 3.1.3. Situational Use Product Associations

**Figure 3:**
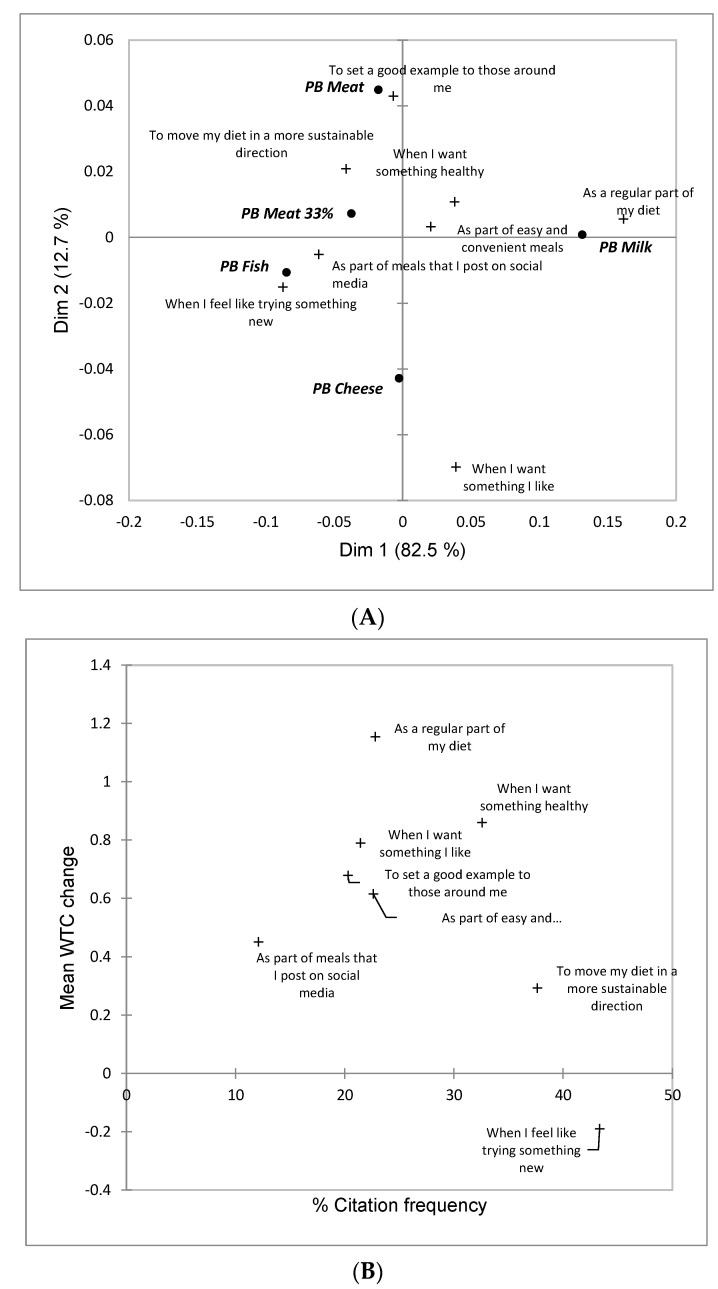
Results linked to situational use product associations for aggregate sample across 2494 consumers in USA, Australia, Singapore and India shown for the five plant-based (PB) food categories included in the research. (**A**) Plot of the first two dimensions following Correspondence Analysis; (**B**) Impact on average willingness to consume (WTC) based on Penalty/Lift analysis. In (**A**), PB food categories are shown in bold and italic font. In (**B**), change in WTC is significantly different from zero (*p* < 0.05) for all terms.

For situational use associations, the PB food categories were significantly differentiated (*p* < 0.05) according to Cochran’s Q tests (except for ‘as part of meals that I post on social media’, *p* = 0.18). Across all stimuli, the most frequent situational use associations were ‘when I feel like trying something new’ (43%) and When I want something healthy’ (33%), while the least frequent association was ‘as part of meals that I post on social media’ (12%). Figure 3A shows a biplot of the two first dimensions after CA, with a dominating first dimension (82.5%) (Part S7 of Supplementary Materials shows the average stimulus positions with 95% confidence intervals). *PB Milk* was separated from the other four PB food categories on Dimension 1 and was the PB category most strongly associated with ‘as a regular part of my diet’ and least frequently associated with ‘when I feel like trying something new’. The second dimension (12.7%) separated *PB Meat* and *PB Cheese*, and the former was significantly more frequently associated with ‘to set a good example to those around me’ and significantly less associated with ‘when I want something I like’.

The Penalty/Lift analysis (Figure 3B) revealed that a positive WTC change greater than one scale point was only found for ‘As a regular part of my diet’. Smaller positive, but still significant (*p* < 0.05) changes in WTC were found for all other situational use situations. The exception was ‘When I feel like trying something new’. On average, selection of this use situation reduced WTC by about 0.2 WTC scale points.

### 3.2. Consumer Segmentation Based on Willingness to Consume

Directed by Objective 2, hierarchical cluster analysis of WTC ratings was performed on the total sample of 2494 people across the four countries. Based on the dendrogram (Part S8 of Supplementary Materials), a 7-cluster solution was retained. With the way of constructing the clusters being ascending, the dendrogram showed that the first notable “jump” in within-cluster variation took place between a 7-cluster and a 6-cluster solution. Moreover, the number of participants in each of the retained clusters was greater than 100, which ensured that the clusters were both homogeneous and large.

Among the 7 retained clusters there was one large cluster (1382 people; 55.4% of total sample) which could be described as PB category non-discriminators. Additionally, there was six smaller clusters (127 to 296 people per cluster; 44.6% of total sample) with distinct patterns of WTC ratings for the different PB food categories. Because the individual clusters had nuanced and complex WTC profiles for PB food category, arbitrary cluster naming (Cluster 1 to Cluster 7) was used to simplify the presentation of results and retain focus on the existence of multiple minor clusters rather than their specific individual WTC profiles.

The demographic profiles of the 7 clusters are summarized in Table 1. There were few between-cluster differences in relation to biological sex, age group or level of education. Participants from India were notably overrepresented in Cluster 2 (63.8%), and to a lesser degree in Cluster 4 (44.6%). Other country differences among clusters were minor, with the percentage distributions ranging between 10% and 34% (relative to 25% for even distributions by country). When considering self-declared dietary preferences, omnivores were overrepresented in Clusters 1, 3 and 7 (60–68%). Flexitarians were more evenly distributed, but with a tendency to higher representation in Clusters 4 and 6 (46–48%). Compared to the overall sample, vegans were strongly overrepresented in Cluster 2 (29%), which is likely attributable to the high percentage of consumers from India falling into Cluster 2. Cluster differences for FN, FTN and environmental concern were minor and not clearly related to WTC for PB food categories.

#### 3.2.1. PB Category Non-Discriminators: Cluster 1

In the retained solution, there was one large group of consumers (55.4%, *n* = 1382) whose WTC responses revealed these people to be PB category non-discriminators. While significantly different, the average WTC ratings for the five PB food categories in this cluster were very similar and the effect size was ‘nil’ (Cluster 1, Table 2).

A follow-up cluster analysis identified three sub-groups of consumers within Cluster 1 (Part S9 of Supplementary Materials includes a dendrogram and a plot of WTC means). Although each of these three sub-groups were non-discriminating among food categories, they differed significantly in the magnitude of their stated WTC. The largest group (*n* = 865, 34.7% of total sample) gave low WTC ratings to all five PB food categories (between ‘2–3 times a year’ and ‘never or less than once a year’). The smallest group (*n* = 203, 8.1% of total sample) gave higher WTC ratings across food categories (between ‘once every month’ and ‘every 2–3 months’), while the third group (*n* = 314, 12.6% of total sample) gave high WTC ratings to all PB food categories (around ‘2–4 times a week’).

Part S10 of Supplementary Materials has the demographic profiles for the Cluster 1 sub-groups and it fits that Group 2 which had the lowest average WTC across the five PB food categories (between ‘never or less than once yearly’ and ‘2–3 times a year’) comprised more older people (60%), more people with lower educational attainment (46.5%), was dominated by self-declared omnivores (73.4%) and was most food neophobic (38.4) and food technology neophobic (60.2). This was contrasted with Group 3 where average WTC was highest. In this group, people from the younger age group (18 to 45 years old) were in the majority (65.3%), as were those who had higher educational attainment (70.7%). Consumers from India were also relatively overrepresented (39.2%) in this latter group.

#### 3.2.2. PB Category Discriminators: Clusters 2 to 7

**Figure 4:**
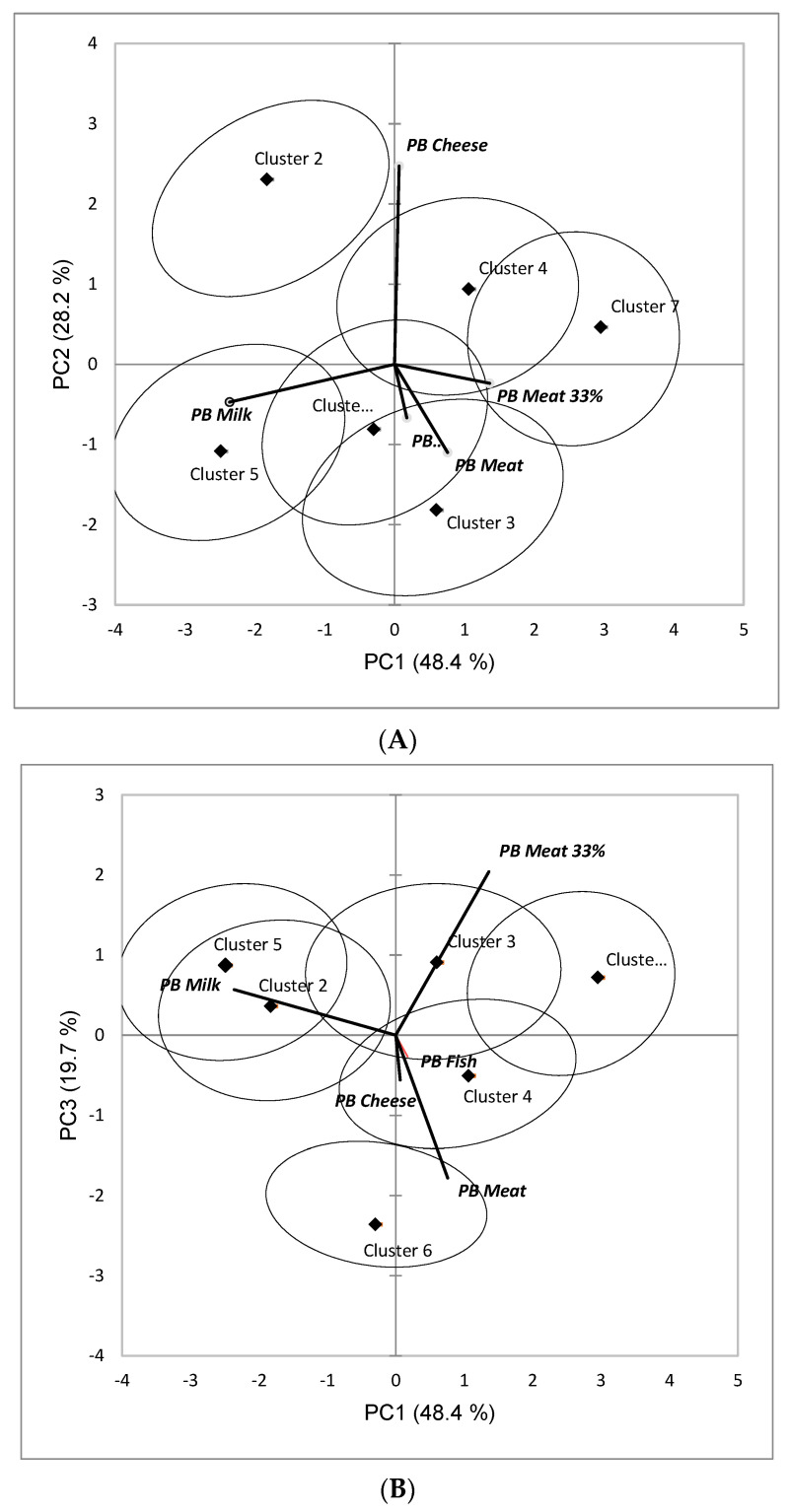
Two-dimensional biplots following Principal Components Analysis of mean willingness to consume (WTC) ratings for PB food categories in the six PB category discriminating clusters (Cluster 2 to Cluster 7). Confidence ellipses (95%) around average cluster positions were obtained by bootstrapping. (**A**) PC1 vs. PC2; (**B**) PC1 vs. PC3. (**A**,**B**), PB food categories are shown in bold and italic font.

Each of the 6 smaller segments had distinct WTC patterns by PB food category, and based on effect size, the PB category differences were largest in Cluster 5 (η^2^ = 0.72) and Cluster 2 (η^2^ = 0.68) (Table 2). In Cluster 5 (*n* = 150, 6.0% of total sample), average WTC ratings were high for *PB Milk* (between ‘2–4 times per week’ and ‘5–6 times per week’) and much lower for all other PB food categories. Among these consumers, *PB Cheese* had the highest WTC, followed by *PB Meat 33%* and then *100% PB Meat* and *PB Fish*. In Cluster 2 (*n* = 127, 5.1% of total sample), the high average WTC for *PB Milk* was similar to Cluster 5. Furthermore, in this cluster WTC for *PB Cheese* was also high (between ‘once every week’ and ‘2–4 times every week’). In a further parallel to Cluster 5, *PB Meat 33%* was the PB food category with the third highest average WTC rating (between ‘every 2–3 months’ and ‘2–3 times a year’). *PB Meat* and *PB Fish* had the lowest average WTC (around ‘2–3 times a year’). These distinct WTC profiles are seen in Figure 4 which presents two-dimensional biplots following Principal Components Analysis of mean WTC ratings by PB product category across the six category discriminating clusters (Figure 4A plots PC1 vs. PC2; Figure 4B plots PC1 vs. PC3). Briefly, PC1 and PC2 separated, respectively, *PB Milk* and *PB Cheese* from the other PB food categories, while PC3 separated the two variants of *PB Meat*.

Cluster 6 (*n* = 143, 5.7% of total sample) resembled Clusters 2 and 5 by having high average WTC ratings for *PB Milk* and *PB Cheese* (Table 1), as well as high WTC for *PB Meat* (between ‘2–4 times per week’ and ‘5–6 times per week’) (Table 2). Conversely, in this cluster, WTC was lowest for *PB Meat 33%* (between ‘every 2–3 months’ and ‘2–3 times a year’), although still higher than in Clusters 2 and 5. Overall, Cluster 6 comprised the people who gave the highest average WTC ratings to PB foods.

In Cluster 3 (*n* = 214, 8.6% of total sample), WTC for *PB Milk* was again high (Table 2), and Cluster 3 resembled Cluster 5 in having a much lower average WTC rating for *PB Cheese* than *PB Milk* (Figure 4A). Differentiating its WTC profile from the other clusters was a higher WTC for 33% *PB Meat* compared to *100% PB Meat* (Figure 4B). Finally, Cluster 3 resembled Cluster 6 in having an overall high WTC for PB foods (Table 1).

Cluster 7 (*n* = 182, 7.3% of total population) had the lowest WTC for *PB Milk* among the six category discriminating clusters (Part S11 of Supplementary Materials has results from ANOVA for each of the five PB food categories separately). Consumers in this cluster rated, on average, WTC for *PB Meat 33%* the highest (between ‘once every month’ and ‘every 2–3 months’). *PB Meat* had the same average rating as *PB Milk* (Table 2). The distinct WTC profile for Cluster 7 was clearly seen in Figure 4.

Cluster 4 was the largest of the six category discriminating clusters (*n* = 296, 11.9% of total sample) and, compared to the other five clusters, differences in mean WTC for the PB food categories were smallest (η^2^ = 0.14) (Table 2), which could also be seen from its position closer to the origins of the plots in Figure 4. The WTC profile in Cluster 4 most strongly resembled that from the aggregate sample (Figure 1), with highest WTC for *PB Milk* followed by *PB Cheese*, and then lower ratings for the other three PB food categories (Table 2).

### 3.3. Emotional, Conceptual and Situational Use Drivers of Willingness to Consume in PB Category Discriminating Consumer Segments

Directed by the third objective of the research (Obj. 3), Penalty/Lift analysis was performed to identify emotional, conceptual and situational use drivers of WTC in each of the PB category discriminating consumer segments (Cluster 2 to Cluster 7). Supported by the findings from Penalty/Lift analysis, where none of the explanatory CATA terms strongly impacted WTC (change in WTC was with one exception always less than 0.5 scale points), no further consideration was given to the non-discriminating PB category cluster (Cluster 1).

#### 3.3.1. Emotional and Conceptual Drivers of WTC by Consumer Segment

The results following Penalty/Lift analysis for PB food categories within each of the six discriminating clusters are shown in Table 3 for the 20 emotional and conceptual terms. When term selection was associated with a change in WTC that was significantly different from zero (*p* < 0.05), the five terms with negative valence—‘boring’, ‘dissatisfied’, ‘nervous’, ‘tense’, and ‘uninspired’—overwhelmingly reduced WTC. Table 3 shows this by the red shading of cells and a negative value up to one WTC scale points (blank cells in Table 3 indicate that significance testing of change in WTC was not performed due to infrequent term use (<5%) for a specific PB food category; tables of citation frequencies for terms by PB food category in individual clusters are given in Part S12 of Supplementary Materials). The two exceptions to this pattern of WTC penalties were Cluster 2 and Cluster 3, where an association between ‘nervous’ and *PB Meat 33%* resulted in a significant WTC increase (Table 3).

Differences between the PB food categories regarding the penalty on WTC of negative emotional associations were seen in Table 3, with reduced WTC for *PB Milk* often associated with the terms ‘boring’ and ‘uninspired’. Conversely, ‘dissatisfied’ was most often associated with reduced WTC for *PB Cheese*. Table 3 also showed that many negative emotional associations reduced WTC for *PB Milk* in Cluster 7 and the same applied to *PB Cheese* in Cluster 6. The average negative impact on WTC was highest for *PB Milk* in Cluster 7, which could tentatively be explained by the low average WTC for this food category in this cluster (Table 1). The largest WTC penalty (1 scale point) was observed for ‘uninspired’ in response to *33% PB Meat* in Cluster 6 and is likely explained by the much lower WTC for this hybrid PB category than the other PB food categories in this cluster. Just as it was an infrequent emotion at the aggregate level (Section 3.1.2), ‘tense’ was infrequently used for individual food categories within clusters and was often <5% (blank cells in Table 3). The only instance where selection of this term was associated with a significant WTC penalty was for *PB Fish* in Cluster 4.

When citation of terms with positive valence (‘enthusiastic’, ‘comforting’, ‘easygoing’, ‘energetic’, ‘happy’, and ‘inspiring’) significantly impacted WTC, the change was always positive, as seen from the green shading in Table 3. The results aligned well with the WTC changes for terms with negative valence. For example, WTC significantly increased when ‘inspiring’ was used to characterize *PB Meat 33%* in Cluster 6, which fitted with the significant WTC decrease for ‘uninspired’ for this product-by-cluster combination. For *PB Cheese* in Cluster 5, significant WTC increases for ‘happy’ and ‘enthusiastic’ fitted with significant WTC decreases for, respectively, ‘dissatisfied’ and ‘uninspired’. Another example was *PB Meat* in Cluster 7, where a WTC increase for ‘comforting’ fitted with a WTC decrease for ‘tense’. In the two instances where ‘easygoing’ was significantly associated with a WTC increase, it was for *PB Fish* (Cluster 3 and Cluster 6).

Contrary to the systematic WTC impact for terms with negative or positive valence (respectively, WTC penalty and WTC lift), selection of conceptual terms (‘adventurous’, ‘classy’, ‘feminine’, ‘passive’, ‘powerful’, ‘pretentious’, ‘sophisticated’, ‘unique’, ‘youthful’) largely resulted in WTC increases, although some occasional WTC decreases were noted (a mix of green and red shading in Table 3). In the case of ‘classy’, WTC significantly increased for *PB cheese* in Cluster 2 and Cluster 5. However, in Cluster 3, WTC was penalized when ‘classy’ was associated with *PB Milk*, while it increased when this term was associated with *PB Fish*. The term ‘feminine’, was associated with a WTC increase for *PB Fish* in Cluster 6, but a WTC decrease for *PB Meat*. Furthermore, in Cluster 6, mixed WTC impacts were seen for ‘pretentious’, which was negative for *PB Meat* but positive for *PB Fish* and *PB Cheese*.

For ‘adventurous’, ‘powerful’ and ‘unique’, WTC changes were positive (green shading), although never for the same clusters and PB food categories. However, in Cluster 7, where *PB Milk* had the lowest within-cluster WTC rating, selection of ‘adventurous’ increased WTC. When ‘powerful’ was significantly associated with WTC increase it was only for *PB Meat* or *PB Meat 33%*. Perceiving PB food categories as ‘sophisticated’ never resulted in a significant WTC change. Least significant impacts on WTC for emotional and conceptual terms were observed for Cluster 4, which was the largest of the six PB category discriminating clusters (11.9% of total sample). There were only two instances (Table 3), which both revealed a WTC penalty – *PB Cheese* (highest WTC in cluster) when associated with ‘boring’, *PB Fish* (lowest WTC in cluster) when associated with ‘tense’.

#### 3.3.2. Situational Use Drivers of WTC by Consumer Segment

Table 4 pertains to situational use terms but is otherwise the same as Table 3. It, shows the impact on WTC for PB food categories in each of the six plant-based (PB) category discriminating clusters (Cluster 2 to Cluster 7) as determined by Penalty/Lift analysis (Part S13 of Supplementary Materials has citation frequency for situational use CATA terms by cluster for the five plant-based (PB) food categories). Compared with the WTC changes observed for emotional and conceptual terms, the average impacts were smaller for situation use situations.

Selection of ‘when I want something I like’ was associated with significant WTC increases in Clusters 3, 4, 6 and 7, but never pertained to *PB Meat 33%*. For the situational use ‘as part of easy and convenient meals’, the significant WTC increases only pertained to *PB Fish* (Clusters 3 and 6). The greater familiarity of *PB Milk* and *PB Cheese* compared with the other PB food categories likely explained why selection of ‘when I feel like trying something new’ was only significant in connection with these two PB food categories and negatively impacted WTC. Selection of ‘when I want something healthy’ resulted in eight significant WTC changes (6 were increases and 2 were decreases), both across and within clusters and across and within PB food categories. In the case of *PB Cheese*, the WTC impact was positive in Cluster 2, 3 and 5 and negative in Cluster 4. In Cluster 7, there was a significant WTC lift for *PB Meat*, but a significant WTC penalty for *PB Meat 33%*. There was also a mix of significant WTC increases and decreases for ‘as a regular part of my diet’ across clusters and PB food categories. There were mostly positive WTC changes associated with selection of ‘to set a good example to those around me’, except for Cluster 4 where a significant lift was found for *PB Meat* but a significant penalty for *PB Cheese*.

Two situational uses – ‘to move my diet in a more sustainable direction’ and ‘to set a good example to those around me’ – were never associated with significant WTC changes.

## 4. Discussion

### 4.1. Willingness to Consume, Emotional, Conceptual, and Situational Use Characterizations for PB Food Categories (Aggregate Level)

About 2500 consumers in four countries took part in the present research, and rated WTC for five categories of PB foods – *PB Milk, PB Cheese, PB Meat, PB Meat 33%* and *PB Fish*. Across all countries and participants, WTC declined in the order as listed above, which separated the “dairy” categories from the “meat” and “fish” categories (Figure 1A). This finding was consistent with initial expectations that the food category to which a product belongs has an important influence on consumer responses to alternative proteins, and the contraposition of PB dairy foods with PB fish was consistent with projective mapping data that show PB dairy products on a perceptual dipole with PB fish products [127].

More generally, the ordering of the PB food categories according to WTC was consistent with the established market for PB milk being the most developed (Good Food Institute, 2022) and, therefore, most familiar to consumers. In contrast, existing challenges with product quality for PB cheese [128,129] and PB meat alternatives [130,131] has hampered migration of these products into the mainstream of consumer purchases. Lastly, PB fish is still extremely novel and not generally available to consumers. The category of fish also suffers from a relatively low level of product interest due to generally lower acceptance of fish products due to sensory, preparation and cost barriers [132,133,134,135].

The average WTC ratings for all PB food categories were low and between ‘1–3 times per week’ and ‘every 2–3 month’ (Figure 1A), which corresponded with low citation frequencies of positive emotional associations (<25% on average) (Part S6 of Supplementary Materials). These findings were consistent with the observed low uptake of PB food categories in general [109] but are concern for the needed transition to more sustainable food systems and consumption patterns [14]. As previously noted by, for example, [51,136], findings ways to help consumers to incorporate PB food categories into their diet is paramount. The present results confirmed this by showing that the situational use ‘as a regular part of my diet’ was associated with the strongest positive impact on WTC in the Penalty/Lift analysis (Figure 3B), followed by associations with specific benefits related to health (‘when I want something healthy’) and taste (‘when I want something I like’).

With this in mind (also known as, helping consumers to find ways to make PB foods a regular part of their diets), it was encouraging that emotional associations to the PB food categories were more positive than negative (Part S6 of Supplementary Materials). Thus, negative overall attitude to PB foods may not be the primary consumption barrier. This may instead lie with product and category specific factors which is an encouraging finding, since such factors can be addressed through the combined efforts of product development, information/education efforts and marketing initiatives [137,138,139]. The conceptual associations supported the notion that marketing and branding efforts can play a role in increasing uptake since significant impacts on WTC were seen for ‘sophisticated’, ‘feminine’, ‘powerful’, ‘classy’, ‘youthful, ‘comforting’ (Figure 3B). Shaping perceptions of PB food categories in positive ways through such conceptual associations can help to overcome negative stereotypes related to early market introductions of poorer quality plant-based products, as described by Cardello et al. [48] in the case of PB milk.

Associations to ‘adventurous’ and ‘unique’ were, on average, also found to positively impact WTC, and as above, branding and product positioning can capitalize on these associations, although care should be taken to ensure that such market positioning does not evoke conceptualizations of extreme novelty that may evoke neophobic responses. This is the case, because consumers with higher levels of food neophobia are known to be laggards in the uptake of PB foods [69,140,141,142], and further associating such foods to novelty might slow their uptake among this group of consumers. More generally, harnessing the novelty of PB food categories as a pathway to greater consumption also seems questionable given the finding that WTC was slightly, but significantly reduced (~0.2 scale points) with the selection of the situational use ‘when I feel like trying something new’. This suggests an expectation that unfamiliar PB foods may not provide enjoyable sensory experiences. Such negative expectations have been raised previously with regard to PB foods [48,143], although as the development of PB foods progresses and products of higher sensory and hedonic quality appear in the market, such negative expectations are likely to diminish.

### 4.2. Consumer Segmentation Based on Willingness to Consume Different PB Food Categories

#### 4.2.1. Consumer Segments Based on WTC for PB Food Categories

The average WTC ratings for the PB food categories masked significant heterogeneity in ratings from individual consumers (Figure 1B) and a 7-cluster solution was derived comprising one large PB non-category discriminating cluster (~55% of participants) and six smaller PB category discriminating clusters (~45% of participants). It was a key finding of the present research that about half of the participants viewed all PB food categories similarly be it relatively positively (with higher WTC ratings) or quite negatively (with low/lower WTC ratings)—while the remaining participants displayed varying preferences (as proxied by WTC) for the different PB food categories. To our knowledge, it has not previously been shown within the context of a wide range of food categories that consumers hold highly specific PB food category preferences, and that, for these people, a high WTC for one PB food category is an unreliable predictor of WTC (and preferences) for other PB food categories. This insight has implications for the promotion of PB diets, since it implies that generic marketing efforts to improve PB consumption may not have their desired effect, because consumers are more or less open to PB foods within one or more food categories, but not others. Future research is needed to explore this possibility and better understand whether consumers are receptive for persuasive arguments regarding increasing PB food consumption for categories where they have low WTC.

While the six PB discriminating clusters (Clusters 2 to 7 in Table 2) had distinct WTC profiles (Figure 2), they also showed consistency, in that *PB Milk* always had the highest/second highest WTC rating, while *PB Fish* always had the lowest/second lowest WTC rating. This corresponded with the aggregate sample WTC results discussed in Section 4.1. The greatest between-cluster differences in WTC were seen for *PB Cheese*, *PB Meat* and *PB Meat 33%,* which in individual clusters varied between having the highest and/or lowest WTC ratings. The PB category discriminating clusters also differed in overall WTC for PB foods, as seen in the average WTC ratings in the second column of Table 2. Clusters 4 and 6 had similar high overall WTC, but this was underpinned by different WTC for 4 of the 5 individual PB food categories (except *PB Fish*) (Part S8 of Supplementary Material has post hoc cluster comparisons of WTC within PB food categories). In Clusters 4 and 6, the greatest share of people who self-declared as flexitarians (46–48%) were found (Table 1). This was in sharp contrast to Cluster 2 which had the lowest overall WTC and “rejected” PB food categories other than *PB Milk*. The highest proportion of self-declared vegans/vegetarians were found in this cluster (29%), confirming previous reports that many PB foods lack appeal for such consumers [37]. The overall WTC in Clusters 3 and 7 was equally low (about ‘once a month’ on average), but this was underpinned by significant differences in WTC for 3 of the 5 PB food categories (*PB Milk, PB Cheese* and *PB Fish*; Part S8 of Supplementary Material). The low overall WTC fitted with the self-declared dietary preferences, which was dominated by omnivores (60–65%) and the near absence of veg*ns (1–4%) (Table 1).

The drivers of the uncovered cluster specific PB food category preferences is likely multifactorial, and also an interactive function of personal cognitive, experiential and value-based associations with PB foods and the specific food categories used in this study. For example, *PB Milk* had highest WTC among most clusters, but it was also perceived as healthier and tastier than most of the other PB food categories according to the situational appropriateness data (Part S10B of Supplementary Materials). While consumers may believe that all PB foods are equally sustainable, these environmental benefits may not lead to an increased WTC, unless other benefits (sensory, health) are also associated with some of the less familiar food categories, like *PB Fish*. Similarly, previous disappointing experiences with PB foods within a specific category, e.g., PB cheeses, can disrupt any generalized halo effect across categories. Likewise, it is possible to envisage consumers making the inference that that if they dislike, e.g., fish, they will also likely dislike PB fish, and in the case of *PB Meat 33%*, it is possible to imagine that people may eschew such a product with the reasoning that they prefer to just eat less meat than eating a PB blend.

By gaining a better understanding of the preference drivers within each cluster, it becomes possible to understand the unique profiles more fully and how to achieve greater PB food uptake. This could take many forms, including a focus on increasing consumption for the PB food category with lowest WTC or focusing on achieving a complete substitution to PB alternatives in the food category where WTC was highest, or combinations hereof. Suggestions like these resonate with previous authors including Lang [136] who note that understanding consumer context and motivations for adopting PB foods has important consequences for marketing positioning, messaging and promotion.

#### 4.2.2. Emotional, Cognitive and Situational Use Drivers of WTC in PB Food Category Discriminating Clusters

Systematically, across all PB food categories and clusters, WTC decreased significantly when associations were made to negative emotions. Similarly, WTC increased significantly when associations were made to positive emotions. However, based on number of significant WTC changes (Table 3), an overall positive effect on WTC was less certain. This could suggest that care must be taken to avoid negative emotions for PB product consumption experiences and understanding that, if they occur, their impact may be greater, in accordance with prospect theory where “losses loom larger” [144,145].

An association between ‘nervous’ and *PB Meat 33%* resulted in a significant WTC increase in Clusters 2 and 3 (Table 3. At a first glance this result was counter intuitive in the sense that a negative emotion would be associated with greater WTC. However, it is possible that consumers in these two clusters were positive about such meat hybrids being available in the marketplace, while at the same time experiencing some nervousness and unease about them because they do not understand what they are. For example, the stimulus description used in this study (*Meat—blend containing 33% plant-based ingredients*) lacked specificity about the source of the non-PB part of the blend. In spite of years of development efforts on hybrid meat products, e.g., PB-extended hamburger products [146], sensory quality issues remain. Grasso and Jaworska [147] who summarized recent commercial developments in the UK in the hybrid meat category noted mixed results in relation to attempts to bring products to market. The authors reported that several early launches had not maintained a place in the market and suggested that hybrids have been received with confusion and are not understood by consumers, leaving them as a minor alternative for those most attached to meat [86]. Desire for ingredient transparency and clean labels by consumers in relation to PB foods [34,148] is also consistent with the need to reduce this continuing confusion with hybrid products.

Health and environmental concern are often regarded as the two key motivators for PB food consumption [109]. In this regard it was interesting that the health-related situational use situation (‘when I want something healthy’) was significantly associated with WTC change in the PB category discriminating clusters, while the environment-related use situation (‘when I want to move my diet in a more sustainable direction’) never was (Table 4). Tentatively both factors are important for motivating PB food uptake, but only personal health concerns are effective at regulating the frequency of consumption. A slightly different interpretation would be that consumers feel that they are actively contributing to the needed sustainability transition if they sometimes eat PB foods, and that as long as they do so sometimes, environmental and biodiversity challenges are not sufficient to motivate increases in PB food consumption. In a study on meat hybrids, Profeta et al. [105] found that choice probability increased with perceived product healthiness, and that this self-centered motive seemed to outperform altruistic motives like animal welfare or environmental concerns when it came to choices within this emerging product category. In a similar vein, Grasso and Jaworska [147] noted that the most recent meat hybrid product launches did not mention flexitarianism and instead stressed flavor, healthiness and convenience, including messages such as “5-a-day”, the convenience of having vegetables already in minced meat and the use of vegetables as flavor enhancers.

Of further interest regarding the role of healthiness in driving WTC for PB foods, was the result in Cluster 7. For the situational use ‘when I want something healthy’ there was a significant WTC lift for *PB Meat*, but a significant WTC penalty for *PB Meat 33%*. Interestingly, the latter had the highest within-cluster WTC, suggesting good intention to consume, but not because of a positive health perception of this hybrid product. None of the other situational uses were associated with significant WTC increases for this cluster-product combination suggesting that other drivers likely exist which explain reasons for consumption or lack thereof. A worthy extension of the present research would be to include more PB category consumption drivers and to disregard those not having relevance. The use ‘as part of meal that I post on social media about’ did not provide useful insights, suggesting that “bragging” about PB food consumption is not something to which consumers in this sample aspired. Had the sample comprised more young people, then social media use may have been cited more frequently, because use of social media is known to decline with age [149].

The result from Cluster 4 further supported the notion that the situational uses included in the present research were not fully encompassing of uses and motivations for consumption. Notably, *PB Cheese* had the highest within-cluster WTC (around ‘once every week’), but significant WTC decreases were associated with selection of ‘when I want something healthy’, ‘as a regular part of my diet’, and ‘to set a good example to those around me’. This suggested that these were not reasons that motivated consumption for consumers in Cluster 4.

### 4.3. Limitations and Suggestions for Future Research

It should be noted that the short text presented to all participants prior to the study, although designed to establish a common frame of reference for all participants, may also have had an influence on participant responses. However, any influence of this text on responses would be a random effect, unlikely to differentially influence responses among consumer segments or food categories. In addition, it should be noted that PB food categories extend far beyond those included in the present research. Some of these categories would be interesting to consider further in relation to category vs. product-specific preferences and would add desirable diversity in terms encompassing ready-to-eat foods (e.g., PB pizza), menu dishes (e.g., PB lasagna), condiments (e.g., PB mayonnaise), and desserts (e.g., PB ice-cream). Whether an ice-cream is PB or not may not matter greatly to consumers considering that ice-cream is an indulgence product and sensory quality and enjoyment is paramount [150,151]. However, it is likely that PB meat topping on a pizza is perceived differently from the patty of a PB meat burger or from the PB meat category in general. Previously, in support of the notion that “gastronomic context” matters, Elzerman et al. [49,50] found that meat substitutes are perceived differently depending on their shape (e.g., pieces vs. mince) and the type of meals they are in (e.g., pasta, soup, and salad). Future research should also look to emerging PB categories, notably PB eggs, which is a new growth category in the PB space [152]. The latter products are interesting because of the important nutritional properties of eggs, their multi-functionality in cooking, and the fact that they may have an important impact on overall PB food consumption and dietary health.

There is often considerable interest in the effect of demographic and socio-economic variables on consumer responses. Where testable hypotheses relating to these can be developed we encourage their testing as part of future research.

## 5. Conclusions

It is clear from the present study that the food category to which a plant-based food belongs can be an important factor in the willingness to consume PB alternatives to animal products. In particular, a large number of distinct clusters of consumers with different WTC for PB foods falling into different food categories was observed in the present research. Among these clusters, there also exists a large cluster of consumers who are less differentiating in their WTC PB products, although they differ significantly in the magnitude of their WTC for these products. In general, WTC is greatest among consumers for *PB Milk* vs. *PB Cheese* or *PB Meat* (100% or 33%). *PB Fish* had generally low WTC for all consumer groups. Emotional, conceptual and situational use characterizations of the PB foods by food category, combined with penalty/lift analysis of the effect of these characterizations on WTC, showed significant effects on WTC. Positive and negative emotional associations effected corresponding positive and negative effects on WTC. Specific conceptualizations, like ‘adventurous’, ‘classy’, ‘feminine’, and ‘pretentious’ had important contributions to WTC for specific PB food categories and within specific consumer clusters. Similarly, most situational use characterizations drove WTC positively, e.g., ‘when I want something healthy’ or ‘when I want something I like’, or negatively, e.g., ‘when I feel like trying something new’, within PB food discriminating clusters, although specific PB food category-cluster combinations produced alternative effects. In sum, the data urge caution when interpreting data on plant-based food preferences/WTC without taking consideration of the specific food category to which the PB food belongs. Findings regarding PB food preferences within one food category should not be generalized to other food categories.

## Figures and Tables

**Table 1:** Participant characteristics for aggregate sample and by consumer segments based on willingness to consume (WTC) for plant-based (PB) food categories.

	Aggregate	Cluster 1	Cluster 2	Cluster 3	Cluster 4	Cluster 5	Cluster 6	Cluster 7
N	2494	1382	127	214	296	150	143	182
Country (%)								
Australia	25	27.6	11.8	18.7	13.5	34.7	22.3	34
India	24.7	17.2	63.8	26.2	44.6	30	25.9	14.3
Singapore	25.1	28.4	14.2	25.7	25.7	15.3	18.9	19.8
United States	25.2	26.8	10.2	29.4	16.2	20	32.9	31.9
Biological sex (%)								
Female	50	49.1	52.8	54.2	46.6	59.3	59.4	40.7
Male	50	50.9	47.2	45.8	53.4	40.7	40.6	59.3
Age group (%)								
18–45 y.o.	49.5	47.8	47.2	54.2	51	45.3	55.2	55.5
46–69 y.o.	50.5	52.2	52.8	45.8	49	54.7	45.8	44.5
Education (%)								
High school, vocational or short graduate	36.8	41.2	19.7	32.7	29.7	37.3	25.2	40.1
University education (Bachelor or higher)	62.8	58.4	80.3	66.8	70.3	62	74.8	58.8
Prefer to not answer	0.4	0.4	0	0.5	0	0.7	0	1.1
Dietary preference (%)								
Flexitarian	33.9	29.2	35.4	35.5	45.9	38	48.3	32.4
Omnivore	59.7	68	35.4	60.3	41.9	52	35.7	66.5
Vegetarian	6.5	2.8	29.1	4.2	12.2	10	16.1	1.1
Food Neophobia	36.8	37.4	37.8	35.1	37.2	35.1	35.4	35.7
[M (SD)]	(10.2)	(10.6)	(8.7)	(9.1)	(9.0)	(11.9)	(9.4)	(10.5)
Food Technology Neophobia	56.3	57.5	55.6	54.4	53.7	57.6	53.2	55.1
[M (SD)]	(11.6)	(11.9)	(11.2)	(10.7)	(11.1)	(11.0)	(11.0)	(10.0)
Environmental concern	62.4	60	67.1	64.3	64.6	64.3	66.1	62.4
[M (SD)]	(12.4)	(11.9)	(11.7)	(11.0)	(10.7)	(11.0)	(12.6)	(11.1)

Food Neophobia (FN) scores could range between 10 and 70, with higher scores reflecting higher levels of FN. Values are mean (M) and standard deviation (SD). Food Technology Neophobia (FTN) scores could range between 13 and 91, with higher scores reflecting higher levels of FTN. Values are mean (M) and standard deviation (SD). Environmental concern (ENV) scores could range between 12 and 84, with higher scores reflecting more positive attitude toward the environment. Values are mean (M) and standard deviation (SD).

**Table 2:** Mean willingness to consume (WTC) ratings by plant-based (PB) food category for the 7-cluster solution based on the aggregate sample (*n* = 2494).

Cluster	PB Foods (Average)	PB Milk	PB Cheese	PB Fish	PB Meat	PB Meat 33%	*p*-Value (by Cluster)	Effect Size (by Cluster)
1 (55.4%)	3.2 C	3.1 B	3.0 B	3.2 AB	3.4 A	3.2 AB	0.006	0.002
2 (5.1%)	4.1 B	7.3 A	6.4 B	2.2 CD	1.9 D	2.6 C	<0.0001	0.68
3 (8.6%)	4.7 AB	6.6 A	3.4 D	4.3 C	4.1 C	4.9 B	<0.0001	0.19
4 (11.9%)	4.4 AB	5.1 B	5.8 A	3.9 C	3.7 C	3.6 C	<0.0001	0.14
5 (6.0%)	3.0 C	7.6 A	2.3 B	1.7 C	1.7 C	1.9 BC	<0.0001	0.72
6 (5.7%)	4.9 A	6.9 A	5.1 C	3.9 D	6.1 B	2.5 E	<0.0001	0.37
7 (7.3%)	4.1 B	3.9 B	4.9 A	2.4 C	3.9 B	5.5 A	<0.0001	0.20

WTC was measured on a 9-point scale (1 = ’Never or less than once yearly’, 5 = ‘1–3 times per month’, 9 = ‘Once daily or more’). Cluster sizes are given as % of the total sample (*n* = 2494). Standard errors around WTC means were between 0.1 and 0.2 for all clusters and all PB food categories). The last two columns show *p*-value and effect size (η^2^) following analysis of variance within clusters. Results from Tukey’s post hoc tests are shown, and within rows, PB food categories with same capital letters are not significant at the 5% level of significance.

**Table 3:** Results following Penalty/Lift analysis for PB food categories in each of the six plant-based (PB) category discriminating clusters (Cluster 2 to Cluster 7), showing emotional and conceptual drivers of willingness to consume (WTC).

**Prodx** **clus**	**Product**	**Cluster**	**PB categoty**	**Boring**	**Dissatisfied**	**Nervous**	**Tense**	**Uninspired**	**Enthusiastic**	**Comforting**	**Easygoing**	**Energetic**	**Happy**	**Inspiring**	**Adventurous**	**Classy**	**Feminine**	**Passive**	**Powerful**	**Pretentious**	**Sophisticated**	**Unique**	**Youthful**
Milk-2	1. Milk	2	PB Milk		−0.2				0.3	−0.2	−0.4	−0.3	−0.1	−0.2	−0.3	−0.1		0.4	0.1		−0.1	0.0	0.3
Cheese-2	2. Cheese	2	PB Cheese		0.1	0.1	0.0	0.0	0.4	−0.2	−0.2		0.1	0.0	0.1	0.5	0.3	−0.2		0.4	0.0	0.4	0.1
Fish-2	3. Fish	2	PB Fish	0.0	−0.2	0.1	−0.3	−0.2	−0.2	0.0	0.4		0.2	−0.1	0.6	0.3	0.5	0.2	0.0	0.0	0.0	−0.1	−0.2
Meat-2	4. Meat	2	PB Meat	−0.2	−0.2	−0.2	−0.1	0.0	0.0	0.1	0.0		0.1	−0.1	0.3	−0.1	0.1	−0.2	0.1	0.1	0.0	0.3	0.0
Meat33-2	5. Meat33	2	PB Meat 33%		−0.3	0.8	0.4	0.5	−0.3	0.0	0.0		0.0	−0.5	−0.5	0.1	−0.5	−0.2	0.3	−0.4	0.0	−0.1	0.5
Milk-3	1. Milk	3	PB Milk	−0.6	−0.4		−0.3	−0.2	0.0	0.2	0.0	−0.1	0.3	0.0	0.0	−0.6		−0.3	0.0	−0.3	0.3	−0.1	−0.4
Cheese-3	2. Cheese	3	PB Cheese	−0.3	−0.9	0.1	−0.4	−0.6	−0.3	−0.3	−0.1		0.1	0.1	0.1	0.0	0.0	0.0	0.0	−0.1	0.2	−0.1	−0.2
Fish-3	3. Fish	3	PB Fish	−0.2	−0.5	−0.3		−0.6	0.3	0.3	0.6	0.4	0.5	0.0	0.5	0.5	0.5	0.8	0.2	0.3	0.5	−0.1	0.6
Meat-3	4. Meat	3	PB Meat	0.0	−0.3	−0.5	−0.5	0.1	−0.3	−0.4	0.4	0.4	0.1	0.6	0.6	0.3	0.2	0.1	0.5	−0.2	0.1	0.2	0.8
Meat33-3	5. Meat33	3	PB Meat 33%	−0.6	−0.3	0.7		−0.3	−0.1	−0.1	−0.4	0.0	0.0	0.2	0.0	0.2	0.0	0.2	0.9	−0.1	−0.2	0.3	−0.1
Milk-4	1. Milk	4	PB Milk	−0.2	−0.1		−0.3	0.2	−0.1	0.2	0.0	−0.2	−0.1	0.2	0.0	0.0	0.0	0.1	0.1	0.0	0.2	−0.2	0.1
Cheese-4	2. Cheese	4	PB Cheese	−0.5	0.0	0.2		−0.1	−0.3	−0.1	−0.2		−0.2	−0.3	0.0	−0.2	−0.1	0.0	−0.1	0.0	−0.1	0.0	0.0
Fish-4	3. Fish	4	PB Fish	0.0	−0.2	−0.3	−0.6	−0.2	0.1	0.1	0.2	0.3	−0.1	0.0	0.0	0.2	−0.3	0.0	−0.2	0.0	0.1	0.0	−0.1
Meat-4	4. Meat	4	PB Meat	−0.1	0.1	0.2	0.2	−0.1	0.0	0.1	0.1		0.1	−0.1	−0.2	0.2	0.1	0.0	0.2	−0.2	−0.1	0.2	0.0
Meat33-4	5. Meat33	4	PB Meat 33%	−0.4	0.1	0.2	0.2	0.1	0.1	0.2	0.0		0.1	0.1	−0.1	0.1	0.1	0.2	−0.2	−0.1	0.0	0.1	0.0
Milk-5	1. Milk	5	PB Milk		0.0			−0.6	0.2	0.0	0.2	0.1	0.1	0.0	−0.1	−0.4		0.4	0.1		0.0	−0.3	−0.4
Cheese-5	2. Cheese	5	PB Cheese	−0.2	−0.5	−0.2		−0.6	0.9	0.2	0.1		0.8	0.4	0.0	1.2	0.6	0.4	0.2	0.2	0.4	0.1	0.0
Fish-5	3. Fish	5	PB Fish	−0.1	−0.1	0.1		0.1	−0.2	0.2	−0.1		−0.1	−0.1	0.1	0.2	0.1	0.1	0.2	−0.1	−0.1	0.0	−0.1
Meat-5	4. Meat	5	PB Meat	−0.2	−0.2	−0.2	0.3	−0.2	0.1	0.3	0.1		0.1	0.3	0.1	0.1	0.1	0.4	0.1	−0.1	−0.1	0.4	0.3
Meat33-5	5. Meat33	5	PB Meat 33%	−0.2	−0.1	−0.3	−0.1	−0.4	0.2	−0.2	0.0	0.3	0.1	0.2	0.0	−0.1	−0.3	0.2		0.2	−0.2	0.0	−0.1
Milk-6	1. Milk	6	PB Milk	−0.7				−0.2	0.2	0.4	0.3	0.2	0.2	0.4	0.4	0.0		−0.4	−0.1	−0.3	0.1	−0.4	0.0
Cheese-6	2. Cheese	6	PB Cheese		−0.6	−0.6		−0.6	0.4	0.3	0.4		0.7	0.2	0.2	−0.1	0.2	0.2	0.1	0.8	0.0	0.6	0.3
Fish-6	3. Fish	6	PB Fish	0.4	−0.5	−0.7		−0.8	0.5	0.5	0.7		0.3	0.1	−0.2	0.5	0.7		0.2	0.6	0.4	0.6	0.3
Meat-6	4. Meat	6	PB Meat	−0.4	0.2	0.2			0.3	0.1	0.1	0.1	0.1	0.1	−0.1	−0.4	−0.6	−0.4	0.5	−0.5	−0.3	−0.3	−0.5
Meat33-6	5. Meat33	6	PB Meat 33%	−0.4	−0.2	−0.1		−1.0	0.0	−0.2	0.4		−0.1	0.8	0.4	−0.2	−0.5	0.1	0.7	−0.3	0.3	0.1	0.4
Milk-7	1. Milk	7	PB Milk	−0.9	−0.9	−0.9		−0.8	0.2	0.4	0.2	0.3	−0.1	0.1	0.6	0.4		−0.5	−0.1	−0.7	0.5	0.5	0.1
Cheese-7	2. Cheese	7	PB Cheese	−0.5	−0.6	−0.2	−0.2	−0.5	0.0	0.3	−0.1		0.1	0.1	−0.1	0.0	0.1	0.1	−0.1	0.1	0.0	−0.3	0.1
Fish-7	3. Fish	7	PB Fish	0.1	0.2	0.1	0.1	0.2	0.3	0.1	0.4	0.6	0.5	0.2	0.2	0.4	0.4	−0.1	−0.3	0.5	0.3	0.0	0.6
Meat-7	4. Meat	7	PB Meat	0.0	−0.2	−0.2	−0.6	−0.2	0.3	0.8	−0.2	0.5	0.1	0.3	0.0	0.2	0.0	0.1	−0.1	−0.4	0.3	0.6	−0.4
Meat33-7	5. Meat33	7	PB Meat 33%	0.3	0.0	−0.1			0.2	0.3	−0.1		−0.1	0.1	−0.1	0.2	−0.1		−0.3	0.1	−0.2	−0.3	0.1

WTC was measured on a 9-point scale (1 = ’Never or less than once yearly’, 5 = ‘1–3 times per month’, 9 = ‘Once daily or more’). Cell values indicate average WTC change, where red shading used for emotional/conceptual terms with average WTC impact significantly less than zero (*p* < 0.05) and green shading used for terms with average WTC impact significantly less than zero (*p* < 0.05). Blank cells indicate that significance testing of change in WTC was not performed due to infrequent term use (<5%) for a focal PB food category. The presentation order for terms is based on grouping as ‘negative valence’, ‘positive valence’ and ‘conceptual’, shown in three blocks from left to right in the figure.

**Table 4:** Results following Penalty/Lift analysis for PB food categories in each of the six plant-based (PB) category discriminating clusters (Cluster 2 to Cluster 7), showing situational use drivers of willingness to consume (WTC).

**Product**	**Cluster**	**Product**	**When I want something I like**	**When I feel like trying something new**	**To move my diet in a more sustainable direction**	**When I want something healthy**	**As part of meals that I post on social media**	**To set a good example to those around me**	**As a regular part of my diet**	**As part of easy and convenient meals**
1. Milk	2	PB Milk	−0.2	−0.1	0.0	0.1	0.0	−0.1	0.3	0.5
2. Cheese	2	PB Cheese	−0.1	0.1	0.3	0.4	0.2	0.0	0.0	0.3
3. Fish	2	PB Fish	0.1	0.1	−0.3	−0.1	−0.1	0.1	−0.2	0.1
4. Meat	2	PB Meat	0.2	−0.1	0.2	0.1	0.0	0.0	0.1	0.0
5. Meat33	2	PB Meat 33%	−0.2	0.4	0.1	−0.3	−0.2	−0.2	−0.4	0.2
1. Milk	3	PB Milk	0.3	−0.4	0.1	0.1	−0.2	0.0	0.2	−0.1
2. Cheese	3	PB Cheese	0.2	−0.4	0.0	0.5	0.4	0.2	0.3	0.2
3. Fish	3	PB Fish	0.5	−0.1	0.2	0.2	0.4	0.8	0.5	0.5
4. Meat	3	PB Meat	−0.1	−0.1	0.2	0.1	−0.4	0.1	0.7	0.4
5. Meat33	3	PB Meat 33%	−0.1	0.2	0.2	0.2	0.1	0.3	−0.1	0.0
1. Milk	4	PB Milk	0.1	0.0	0.1	0.0	0.1	−0.2	0.0	−0.1
2. Cheese	4	PB Cheese	0.1	−0.1	−0.2	−0.3	−0.3	−0.3	−0.3	0.0
3. Fish	4	PB Fish	0.0	0.1	0.0	−0.2	−0.2	−0.2	0.0	0.1
4. Meat	4	PB Meat	0.3	0.0	0.1	0.1	0.0	0.4	−0.1	0.0
5. Meat33	4	PB Meat 33%	0.1	−0.1	0.1	0.1	0.2	0.2	0.3	0.1
1. Milk	5	PB Milk	−0.1	−0.1	−0.2	−0.1	0.1	0.0	0.2	0.0
2. Cheese	5	PB Cheese	0.3	0.0	0.0	0.5	−0.2	0.4	0.2	0.1
3. Fish	5	PB Fish	−0.1	0.0	−0.1	0.0	0.1	−0.1	0.2	−0.1
4. Meat	5	PB Meat	0.0	0.0	0.1	0.2	0.2	0.4	0.3	0.1
5. Meat33	5	PB Meat 33%	−0.1	0.0	0.1	−0.1	−0.1	−0.3	−0.1	0.0
1. Milk	6	PB Milk	0.1	−0.5	0.0	0.2	−0.2	−0.2	0.5	0.2
2. Cheese	6	PB Cheese	0.5	−0.1	0.0	0.4	0.3	0.8	0.6	0.2
3. Fish	6	PB Fish	0.4	0.3	0.2	0.9	0.0	0.0	0.5	0.6
4. Meat	6	PB Meat	0.1	−0.2	−0.1	−0.2	−0.1	0.3	0.3	−0.3
5. Meat33	6	PB Meat 33%	−0.1	0.2	0.1	0.4	−0.3	0.3	0.2	0.4
1. Milk	7	PB Milk	0.6	−0.1	−0.2	0.7	0.4	−0.1	0.7	0.2
2. Cheese	7	PB Cheese	0.3	−0.1	0.0	0.2	0.3	−0.2	0.0	0.1
3. Fish	7	PB Fish	0.2	−0.1	0.1	0.0	0.3	0.0	−0.2	−0.1
4. Meat	7	PB Meat	0.2	0.2	0.3	0.3	0.3	−0.1	0.2	0.2
5. Meat33	7	PB Meat 33%	−0.3	−0.1	0.0	−0.3	−0.2	−0.1	−0.1	−0.1

WTC was measured on a 9-point scale (1 = ’Never or less than once yearly’, 5 = ‘1–3 times per month’, 9 = ‘Once daily or more’). Cell values indicate average WTC change, where red shading used for emotional/conceptual terms with average WTC impact significantly less than zero (*p* < 0.05) and green shading used for terms with average WTC impact significantly less than zero (*p* < 0.05).

## Data Availability

Data that support the findings of this study are available upon request to the authors.

## Supplementary Materials

The following supporting information can be downloaded at: https://www.mdpi.com/article/10.3390/foods11193059/s1: (1) Summary of participant characteristics by country; (2) List of 12 food technologies and background text given to participants about each of these; (3) Items included in the environmental concern scale; (4) Information regarding online data quality by country; (5) Descriptive statistics and non-parametric tests for the 5 PB food categories (aggregate sample); (6) Citation frequency for emotion, conceptual and situational use terms by for the five plant-based (PB) food categories; (7) Stimuli plots with 95% confidence intervals following Correspondence Analysis (aggregate sample) for emotional/conceptual and situational use responses; (8) Results following hierarchical cluster analysis on willingness to consume (WTC) ratings (7-cluster solution for aggregate sample); (9) Results following hierarchical cluster analysis on willingness to consume (WTC) ratings (3 sub-groups in C1 from aggregate sample); (10) Summary of participant characteristics in the 3 sub-groups of the PB food non-discriminating cluster (Cluster 1); (11) Results of ANOVA of WTC across clusters by PB food category; (12) Citation frequency for emotional and conceptual CATA terms by cluster for the five plant-based (PB) food categories; (13) Citation frequency for situational use CATA terms by cluster for the five plant-based (PB) food categories [153,154].
