# Supplementary material for: Consumer Preference Segments for Plant-Based Foods: The Role of Product Category
Source: Foods. 2022 Oct 1;11(19):3059. doi: 10.3390/foods11193059 (PMC9562706; doi:10.3390/foods11193059)
Supplement: Supplementary file 1 [file foods-11-03059-s001.zip › foods-1923481-supplementary.pdf]

SUPPLEMENTARY MATERIAL

PART S1. Summary of participant characteristics by country with United States (US, n=629), Australia (AU, n=623), Singapore (SG, n=627), and India (IN, n=615).

	US	AU	SG	IN
**Biological sex (%)**				
Female	50.1	50.2	50.1	49.8
Male	49.9	49.8	49.9	50.2
**Age group (%)**				
18-45 y.o.	49.6	49.4	49.4	49.6
46-69 y.o.	50.4	50.6	50.6	50.4
**Education (%)**				
High school, vocational or short graduate	45.6	49.1	39.7	12.4
University education (bachelor or higher)	53.9	49.9	60.3	87.6
Prefer to not disclose	0.5	0.0	0.0	0.0
**Dietary preferences (%)**				
Flexitarian	28.5	22.3	40.0	44.9
Omnivore	68.4	75.4	57.6	36.9
Vegetarian	3.2	2.2	2.4	18.2
**Food Neophobia [M (SD)]**	37.3 (12.4)	39.4 (11.0)	38.3 (8.1)	37.4 (8.2)
**Food Technology Neophobia [M (SD)]**	57.6 (13.0)	56.7 (11.8)	55.8 (9.2)	55.1 (11.8)
**Environmental concern [M (SD)]**	58.9 (14.7)	62.5 (13.4)	62.1 (10.0)	65.9 (9.9)

PART S2. Full list of food stimuli and background text.

1. Milk – from 100% plant-based ingredients
2. Cheese – from 100% plant-based ingredients
3. Vegetables – packaged in a modified atmosphere to increase shelf-life
4. Vegetables – grown indoors in buildings located in cities (urban farming)
5. Fish – reared indoors in buildings where fish and vegetables are farmed together (aquaponics)
6. Fish – from cell cultures grown in a laboratory
7. Fish – from 100% plant-based ingredients
8. Meat – from cell cultures grown in a laboratory
9. Meat – from 100% plant-based ingredients
10. Meat – blend containing 33% plant-based ingredients
11. Meat – blend containing 10% insect powder
12. Bread – from wheat strain transformed by gene-editing to be high-yielding

Our current approaches to food production and our consumption levels are global problems because they drive climate change and environmental degradation. Animal farming is particularly damaging for the environment, and to reduce environmental impacts we must change the way we eat – increasing our consumption of plant foods while substantially limiting our intake from animal sources.

To support this transition, interest has centered on those plant foods that are good sources of proteins – soybeans, nuts, peas, and some grains – and can provide a nutritionally sound alternative to foods from animals, while also being suitable for the growing number of people who do not, or cannot, eat certain animal-sourced foods (e.g., vegetarians/vegans, people who have a dairy allergy or are lactose intolerant).

In the past decade, more and more of these 100% plant-based foods have become available in the United States / Australia / Singapore / India. Plant-based "milks" made from, for example, soy, almonds, oats, and even cashew nuts are fairly familiar now, and plant-based "yoghurt" and "cheese" are no longer uncommon. Plant-based "meat", "fish", and "seafood" are much more novel.

Besides replacing foods from animals with plant-based foods, or creating blends of animal-plant foods, there is also considerable interest in developing alternative sources of animal-derived proteins. These include insects – which are important in some traditional diets around the world – because they can be farmed at large scale with low environmental impact. In particular, "insect flour", which can be used in many different types of foods, is being considered for more widespread consumption.

Laboratory-cultured meat/fish, produced from cultured animal cells, is a potential game-changer from an environmental point of view. There is still some way to go before this way of producing food can feed large numbers of people, but it may become the norm in future.

Alongside these different innovations, there is much interest in improving existing technologies used in food production. These include, for example, improved packaging materials for perishable foods such as fruits, vegetables and bread to improve their shelf-life and require less refrigeration. There are also ongoing efforts to improve crop yields and drought resistance, and these goals can be achieved using gene-editing technologies.

Because the world continues to urbanize, there are also many efforts to develop urban farming. This means producing food in cities rather than in rural districts, which means that transportation is no longer needed, to the benefit of the environment. The most well-known example of this is vertical

farming, where crops are grown indoors, stacked vertically in layers, removing land-use requirements almost entirely. Some vertical farming systems seek to further reduce environmental impacts of food production by recovering nutrients from one food system and using them in another, increasing both efficiency and sustainability. "Aquaponics" – a system where fish and vegetables are reared in the same indoor facility in a way that the nutrient-rich water from the fish rearing supports the plant growth – is one example.

PART S3. Items include in the scale to measure environmental concern. Reverse coded items are denoted with *

**Environmental concern**	**Origin**
When humans interfere with nature it often produces disastrous consequences	Item 3, Dunlap et al. [1]
Humans are severely abusing the environment	Item 5, Dunlap et al. [1]
Despite our special abilities, humans are still subject to the laws of nature	Item 9, Dunlap et al. [1]
If things continue on their present course, we will soon experience a major ecological catastrophe	Item 15, Dunlap et al. [1]
The news media have exaggerated the ecological problem*	Item 1, Lounsbury and Tornatzky [2]
If mankind is going to survive at all, environmental pollution must be stopped	Item 2, Lounsbury and Tornatzky [2]
I am worried about future children's chance of living in a clean environment	Item 4, Lounsbury and Tornatzky [2]
We shouldn't worry about environmental problems because science and technology will solve them before very long*	Item 5, Lounsbury and Tornatzky [2]
It genuinely infuriates me to think that the government doesn't do more to help control pollution of the environment	Item 23, Maloney et al. [3]
I become incensed when I think about the harm being done to plant and animal life by pollution	Item 25, Maloney et al. [3]
When I think of the ways industries are polluting, I get frustrated and angry	Item 28, Maloney et al. [3]
The whole pollution issue has never upset me too much since I feel it's somewhat overrated*	Item 29, Maloney et al. [3]

•

PART S4. Data quality statement.

United States

The final sample of 629 people was reached by approaching a total of 2143 people, of which 469 did not satisfy the eligibility criteria, and 360 began but did not complete the survey. *Post-hoc*, 16 people who had completed the survey were excluded because they had completed the survey very quickly (operationalised as faster than 1/3 of the median response time), which is linked to careless responding [4]. A total of 37 people who completed the survey and passed the "speeder criterion" were also removed *post-hoc* because they provided the same score (i.e., flatlining) [5] for all statements in the 10-item food neophobia scale [6] as well as the 13-item food technology neophobia scale [6]. A further 8 people were removed because they provided random or nonsensical verbatim responses. Of the 1253 qualified participants, 624 people completed other questions that are not related to the present research. The median time to complete the required tasks was 12.0 (IQR=9.3) min.

Australia

The final sample of 623 people was reached by approaching a total of 815 people, of which 122 did not satisfy the eligibility criteria, and 43 began but did not complete the survey. *Post-hoc*, 11 people who had completed the survey were excluded because they had completed the survey very quickly (operationalised as faster than 1/3 of the median response time), which is linked to careless responding [4]. A total of 16 people who completed the survey and passed the "speeder criterion" were also removed *post-hoc* because they provided the same score (i.e., flatlining) [5] for all statements in the 10-item food neophobia scale [6] as well as the 13-item food technology neophobia scale [7]. The median time to complete the required tasks was 12.5 (IQR=8.8) min.

Singapore

The final sample of 627 people was reached by approaching a total of 810 people, of which 109 did not satisfy the eligibility criteria, and 26 began but did not complete the survey. *Post-hoc*, 22 people who had completed the survey were excluded because they had completed the survey very quickly (operationalised as faster than 1/3 of the median response time), which is linked to careless responding [4]. A total of 26 people who completed the survey and passed the "speeder criterion" were also removed *post-hoc* because they provided the same score (i.e., flatlining) [5] for all statements in the 10-item food neophobia scale [6] as well as the 13-item food technology neophobia scale [7]. The median time to complete the required tasks was 12.8 (IQR=11.0) min.

India

The final sample of 615 people was reached by approaching a total of 1004 people, of which 313 did not satisfy the eligibility criteria, and 45 began but did not complete the survey. *Post-hoc*, 19 people who had completed the survey were excluded because they had completed the survey very quickly (operationalised as faster than 1/3 of the median response time), which is linked to careless responding [4]. A total of 11 people who completed the survey and passed the "speeder criterion" were also removed *post-hoc* because they provided the same score (i.e., flatlining) [5] for all statements in the 10-item food neophobia scale [6] as well as the 13-item food technology neophobia scale [7]. A further 1 person was removed because they provided random or nonsensical verbatim responses. The median time to complete the required tasks was 15.5 (IQR=11.4) min.

PART S5. Willingness to consume (WTC) ratings for the five plant-based (PB) food categories included in the study based on the aggregate sample of 2494 consumers across the USA, Australia, Singapore and India. WTC was rated on a 9-pt scale (1='Never or less than once yearly', 5 = '1-3 times per month', 9 = 'Once daily or more').

**Category**	**Mean (SD)**	**Mean of ranks**	**Median (IQR)**
PB Milk	4.4 (2.9)	7105.5 (A)	4 (6)
PB Cheese	3.8 (2.6)	6418.4 (B)	3 (5)
PB Meat	3.5 (2.5)	6039.6 (C)	3 (5)
PB Meat 33%	3.4 (2.5)	5983.7 (C)	3 (4)
PB Fish	3.2 (2.5)	5630.3 (D)	3 (4)

PART S6. Citation frequency (%) for emotional, conceptual and situational use CATA terms (aggregate sample). Results from Cochran Q test (final column) and post-hoc comparisons (Sheskin) for the five plant-based products in the study.

S6A. Emotional and conceptual terms.

Terms	PB Cheese	PB Fish	PB Meat	PB Meat 33%	PB Milk	p-values
Uninspired	12.7 (abc)	14.6 (c)	13.7 (bc)	12.2 (ab)	10.8 (a)	**<0.0001**
Enthusiastic	17.6 (b)	13.9 (a)	15.7 (ab)	14.8 (a)	14.2 (a)	**0.000**
Nervous	7.9 (ab)	11.8 (d)	10.1 (cd)	9.4 (bc)	6.5 (a)	**<0.0001**
Passive	7.5 (a)	7.5 (a)	8.1 (a)	8.7 (a)	8.6 (a)	0.208
Tense	4.9 (ab)	6.4 (bc)	6.7 (c)	6.0 (bc)	4.2 (a)	**<0.0001**
Dissatisfied	14.6 (a)	18.8 (c)	16.9 (bc)	14.8 (ab)	13.6 (a)	**<0.0001**
Adventurous	17.1 (ab)	16.8 (ab)	17.4 (ab)	18.2 (b)	15.3 (a)	**0.032**
Boring	8.6 (ab)	8.7 (ab)	10.0 (b)	8.0 (a)	7.9 (a)	**0.013**
Classy	11.8 (c)	8.5 (ab)	8.9 (ab)	7.3 (a)	10.0 (bc)	**<0.0001**
Comforting	12.9 (b)	8.6 (a)	11.2 (b)	11.1 (b)	20.6 (c)	**<0.0001**
Easygoing	13.4 (bc)	11.3 (ab)	12.8 (abc)	10.7 (a)	14.8 (c)	**<0.0001**
Energetic	4.5 (a)	4.9 (a)	4.9 (a)	4.3 (a)	16.8 (b)	**<0.0001**
Feminine	16.4 (d)	12.8 (bc)	14.4 (cd)	12.1 (b)	4.4 (a)	**<0.0001**
Happy	20.8 (b)	17.9 (a)	20.6 (b)	19.4 (ab)	23.5 (c)	**<0.0001**
Inspiring	15.2 (a)	13.2 (a)	15.4 (a)	14.1 (a)	21.5 (b)	**<0.0001**
Powerful	8.1 (a)	9.9 (a)	9.9 (a)	8.9 (a)	16.1 (b)	**<0.0001**
Pretentious	11.5 (b)	10.7 (b)	10.5 (b)	9.9 (b)	7.0 (a)	**<0.0001**
Sophisticated	24.9 (b)	25.0 (b)	23.9 (b)	23.7 (b)	11.1 (a)	**<0.0001**
Unique	16.8 (b)	13.7 (a)	15.5 (ab)	16.6 (b)	25.1 (c)	**<0.0001**
Youthful	12.7 (c)	9.5 (ab)	11.1 (bc)	8.7 (a)	15.0 (d)	**<0.0001**

S6B. Situational use terms.

Terms	PB Cheese	PB Fish	PB Meat	PB Meat33%	PB Milk	p-values
When I want something I like	24.4 (b)	19.4 (a)	19.4 (a)	20.1 (a)	23.7 (b)	**<0.0001**
When I feel like trying something new	44.5 (b)	45.7 (b)	43.3 (b)	44.7 (b)	38.5 (a)	**<0,.0001**
To move my diet in a more sustainable direction	36.9 (ab)	37.0 (ab)	39.6 (b)	38.1 (ab)	36.5 (a)	**0.023**
When I want something healthy	32.6 (b)	29.0 (a)	33.3 (bc)	31.6 (ab)	36.3 (c)	**<0.0001**
As part of meals that I post on social media	12.6 (a)	12.4 (a)	12.6 (a)	11.6 (a)	11.2 (a)	0.177
To set a good example to those around me	19.8 (a)	18.6 (a)	22.5 (b)	19.9 (a)	20.7 (ab)	**0.000**
As a regular part of my diet	22.3 (b)	18.0 (a)	22.1 (b)	20.4 (ab)	31.1 (c)	**<0.0001**
As part of easy and convenient meals	23.6 (b)	19.5 (a)	23.3 (b)	22.7 (b)	23.9 (b)	**<0.0001**

PART S7. Results for aggregate sample to supplement, respectively, Figure 2a and Figure 3a, showing average stimuli positions with 95% confidence ellipses following Correspondence Analysis. A) Emotional and conceptual stimuli associations; B) Situational use stimuli associations.

(A)

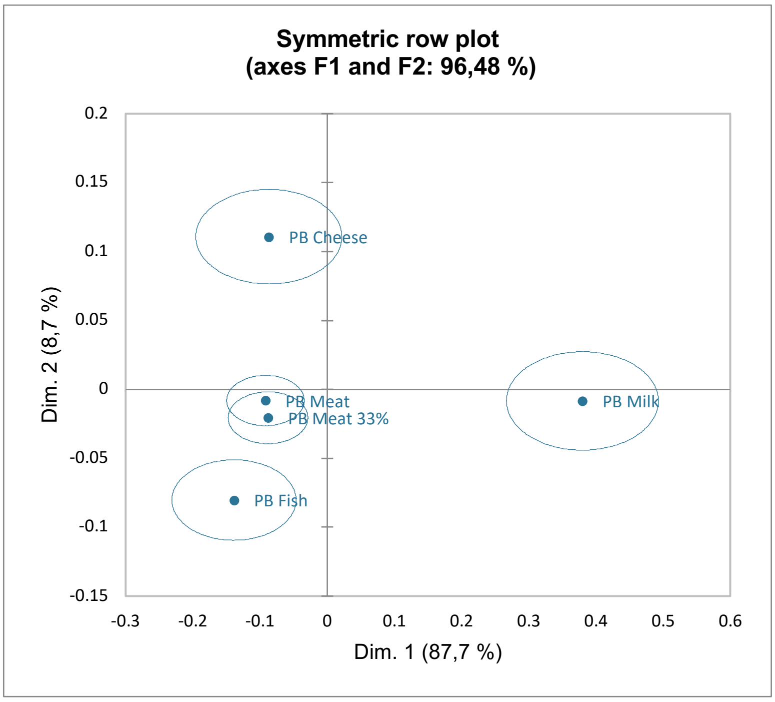

(B)

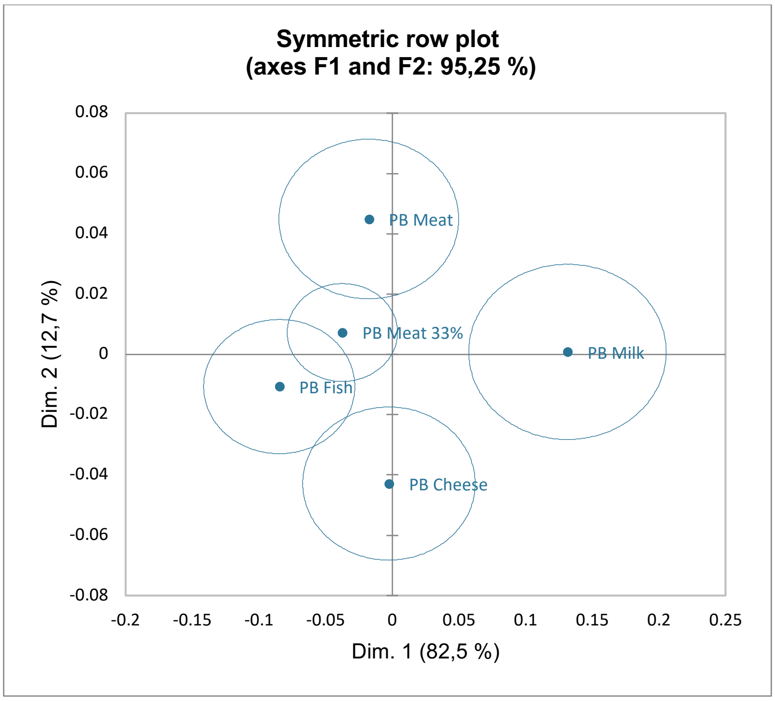

PART S8. Results following hierarchical cluster analysis on Willingness to Consume (WTC) ratings for aggregate sample. A) Dendrogram for 7-cluster solution; B) Mean WTC ratings (1='Never or less than once yearly', 5 = '1-3 times per month', 9 = 'Once daily or more') by cluster for PB food categories (standard errors between 0.1 and 0.2 for all clusters and all PB food categories).

(A)

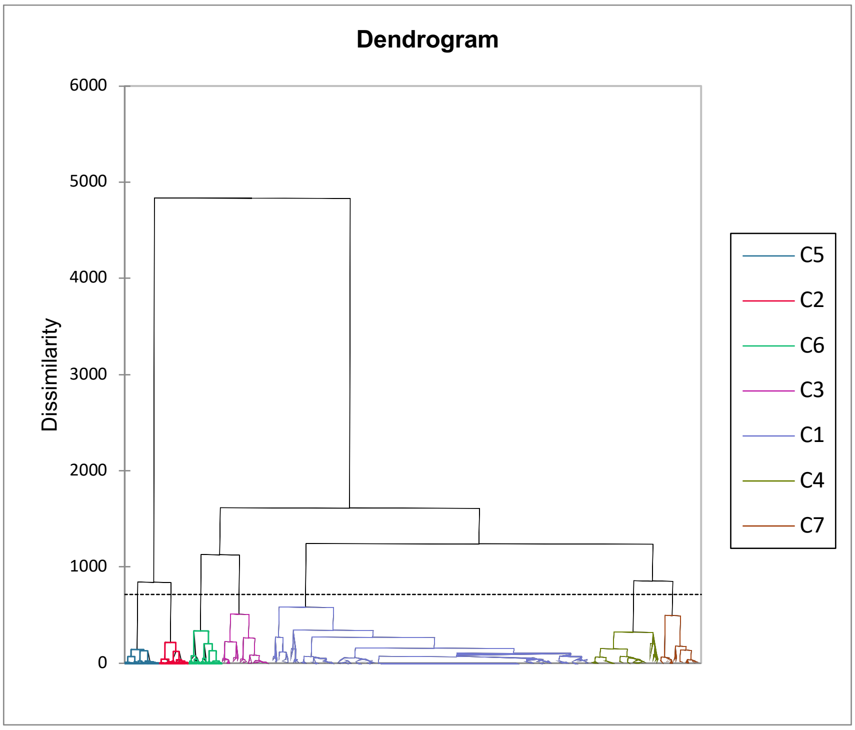

(B)

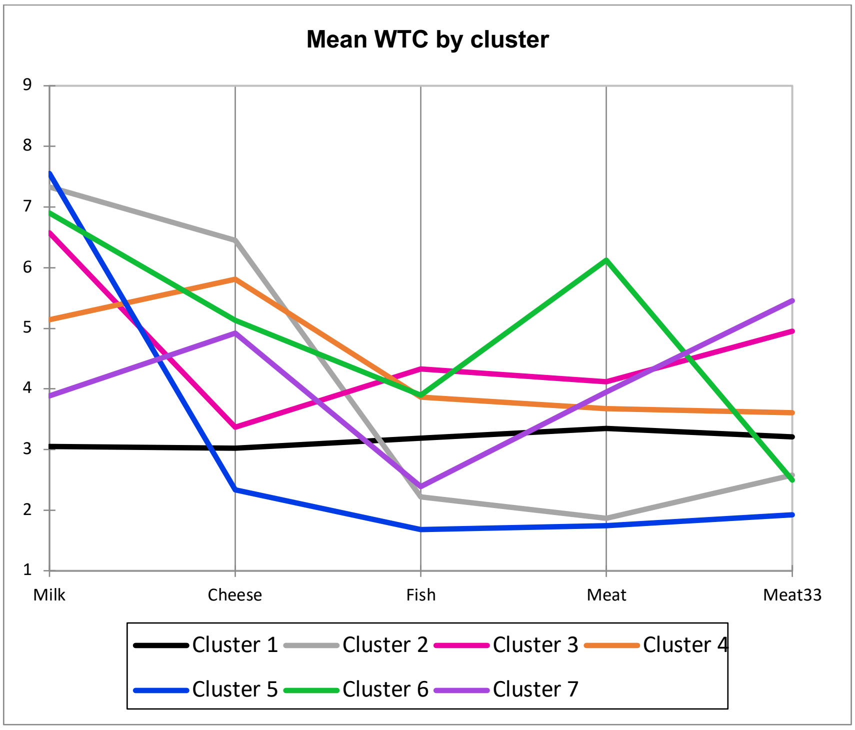

PART S9. Results following hierarchical cluster analysis on Willingness to Consume (WTC) ratings for Cluster 1. A) Dendrogram for 3-cluster solution; B) Mean WTC ratings (1='Never or less than once yearly', 5 = '1-3 times per month', 9 = 'Once daily or more') by cluster for PB food categories (standard errors between 0.1 and 0.2 for all clusters and all PB food categories). The number of consumers in each cluster were: C1=G1=203, C2=G2=865, and C3=G2=314.

(A)

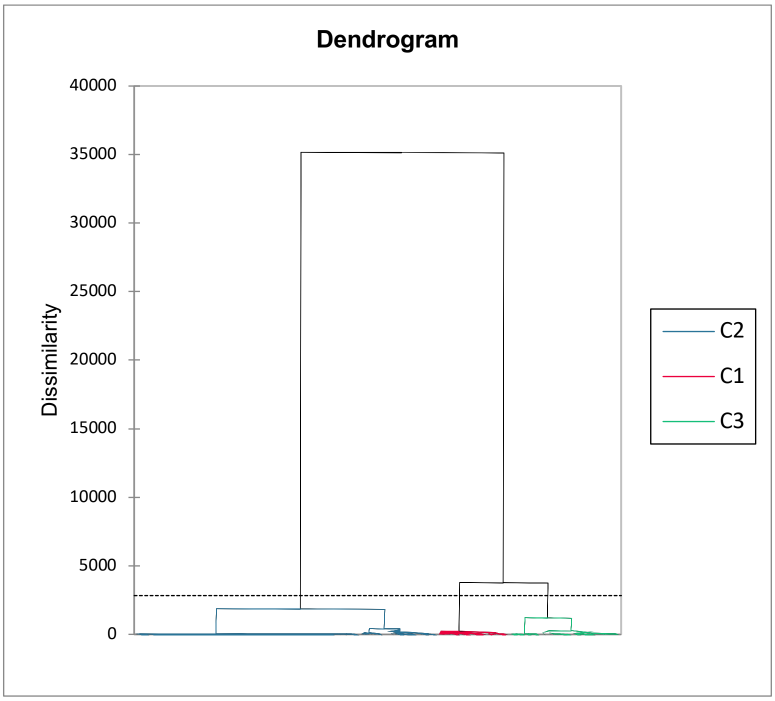

(B)

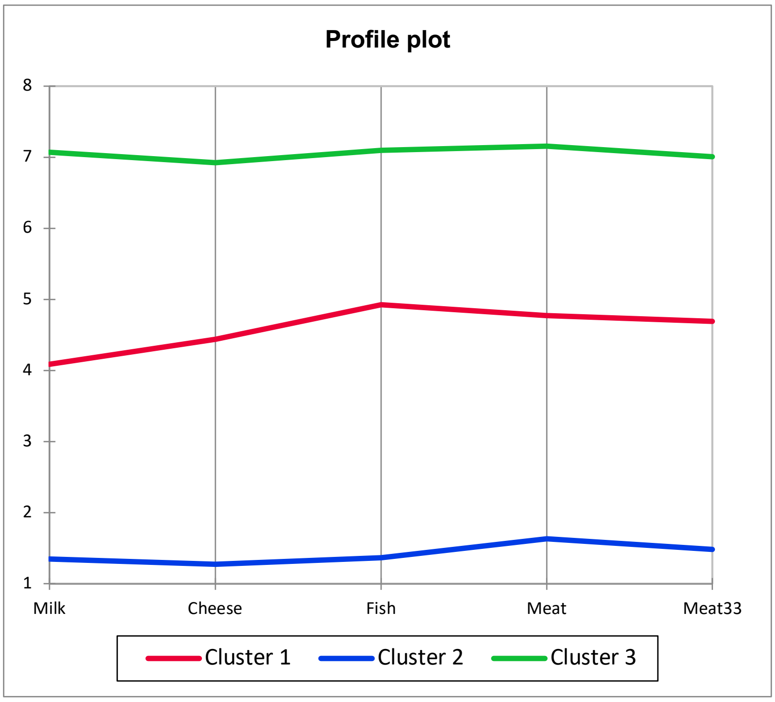

PART S10. Participant characteristics for the three sub-groups in the non-category discriminating cluster (C1) based on willingness to consume (WTC) for plant-based (PB) food categories.

	**Group 1**	**Group 2**	**Group 3**
	N=203	N=865	N=314
	"medium" avg. WTC	"low" avg. WTC	"high" avg. WTC
**Country (%)**			
Australia	19.2	35.2	12.4
India	18.7	8.9	39.2
Singapore	39.4	26.8	25.2
United States	22.7	29.1	22.9
**Biological sex (%)**			
Female	42.9	50.6	49.0
Male	57.1	49.4	51.0
**Age group (%)**			
Younger (18-45 y.o.)	55.2	39.7	65.3
Older (46-69 y.o.)	44.8	60.4	34.7
**Education (%)**			
High school, vocational or short graduate	37.4	46.5	29.3
University education (Bachelor or higher)	62.1	53.0	70.7
Prefer to not answer	0.5	0.5	0.0
**Dietary preferences (%)**			
Flexitarian	37.9	23.8	38.2
Omnivore	58.1	73.4	59.6
Vegetarian	4.0	2.8	2.2
**Food Neophobia [M (SD)]**	36.3 (8.6)	38.4 (11.4)	35.4 (8.8)
**Food Technology Neophobia [M (SD)]**	53.5 (7.7)	60.2 (12.0)	52.8 (11.9)
**Pro-Environmental Attitude [M (SD)]**	61.1 (10.3)	59.5 (14.1)	63.1 (9.5)

PART S11. Mean willingness to consume (WTC) ratings (1='Never or less than once yearly', 5 = '1-3 times per month', 9 = 'Once daily or more') by plant-based (PB) food category for the 6 PB discriminating clusters (n=1112; 44.6% of total sample) (standard errors between 0.1 and 0.2 for all clusters and all PB food categories). The last two rows show p-value and effect size (η^2) following analysis of variance within PB food categories. Results from Tukey's post-hoc tests shown (within columns, clusters with same letters are not significant at the 5% level of significance).

**Cluster (N)**	**PB Milk**	**PB Cheese**	**PB Fish**	**PB Meat**	**PB Meat 33%**
2 (127)	7.3 A	6.4 A	2.2 BC	1.9 C	2.6 C
3 (214)	6.6 B	3.4 D	4.3 A	4.1 B	4.9 A
4 (296)	5.1 C	5.8 B	3.9 A	3.7 B	3.6 B
5 (150)	7.6 A	2.3 E	1.7 C	1.7 C	1.9 C
6 (143)	6.9 AB	5.1 C	3.9 A	6.1 A	2.5 C
7 (182)	3.9 D	4.9 C	2.4 B	3.9 B	5.5 A
P-value	<0.0001	<0.0001	<0.0001	<0.0001	<0.0001
Effect size	.34	.22	.07	.12	.12

PART S12. Citation frequency (%) for emotional and conceptual CATA terms. Results from Cochran Q test (final column) and post-hoc comparisons (Sheskin) by cluster (Cluster 2 to Cluster 7) for the five plant-based products in the study.

S12A. CLUSTER 2.

**Term**	**PB Cheese**	**PB Fish**	**PB Meat**	**PB Meat 33%**	**PB Milk**	**p-value**
Adventurous	16.5 (a)	14.2 (a)	17.3 (a)	18.9 (a)	16.5 (a)	0.860
Boring	3.9 (a)	5.5 (a)	7.1 (a)	3.9 (a)	1.6 (a)	0.186
Classy	5.5 (a)	8.7 (a)	10.2 (a)	7.9 (a)	10.2 (a)	0.486
Comforting	15.7 (ab)	7.9 (a)	12.6 (ab)	10.2 (a)	22.8 (b)	**0.002**
Dissatisfied	7.9 (a)	10.2 (a)	12.6 (a)	9.4 (a)	7.1 (a)	0.434
Easygoing	9.4 (a)	7.1 (a)	8.7 (a)	7.9 (a)	12.6 (a)	0.449
Energetic	4.7 (a)	3.9 (a)	4.7 (a)	2.4 (a)	15.7 (b)	**<0.0001**
Enthusiastic	16.5 (a)	11.0 (a)	15.7 (a)	16.5 (a)	15.0 (a)	0.638
Feminine	17.3 (b)	10.2 (ab)	15.0 (b)	13.4 (ab)	3.9 (a)	**0.002**
Happy	26.0 (a)	26.8 (a)	25.2 (a)	24.4 (a)	32.3 (a)	0.508
Inspiring	11.0 (a)	12.6 (a)	11.8 (a)	10.2 (a)	26.8 (b)	**0.0002**
Nervous	9.4 (a)	11.0 (a)	6.3 (a)	9.4 (a)	3.1 (a)	0.064
Passive	9.4 (a)	9.4 (a)	8.7 (a)	10.2 (a)	8.7 (a)	0.987
Powerful	3.9 (a)	10.2 (ab)	8.7 (ab)	8.7 (ab)	14.2 (b)	0.064
Pretentious	9.4 (ab)	16.5 (b)	18.1 (b)	12.6 (b)	1.6 (a)	**<0.0001**
Sophisticated	22.0 (a)	26.8 (a)	25.2 (a)	26.0 (a)	13.4 (a)	**0.033**
Tense	6.3 (a)	7.1 (a)	9.4 (a)	5.5 (a)	2.4 (a)	0.166
Uninspired	12.6 (a)	10.2 (a)	11.0 (a)	7.1 (a)	4.7 (a)	0.116
Unique	10.2 (a)	10.2 (a)	13.4 (ab)	15.0 (ab)	22.0 (b)	**0.026**
Youthful	8.7 (a)	8.7 (a)	11.8 (a)	6.3 (a)	13.4 (a)	0.208

S12B. CLUSTER 3.

Term	PB Cheese	PB Fish	PB Meat	PB Meat 33%	PB Milk	p-value
Adventurous	22.0 (a)	21.0 (a)	20.6 (a)	22.0 (a)	17.3 (a)	0.659
Boring	8.4 (a)	7.5 (a)	7.5 (a)	6.1 (a)	5.1 (a)	0.564
Classy	13.6 (a)	8.9 (a)	11.2 (a)	8.4 (a)	8.4 (a)	0.169
Comforting	14.5 (ab)	7.5 (a)	12.1 (a)	9.3 (a)	20.6 (b)	**<0.0001**
Dissatisfied	12.6 (b)	11.2 (ab)	11.2 (ab)	10.3 (ab)	5.1 (a)	**0.042**
Easygoing	15.4 (a)	12.1 (a)	14.0 (a)	12.1 (a)	15.4 (a)	0.616
Energetic	1.4 (a)	7.0 (a)	7.0 (a)	7.5 (a)	22.9 (b)	**<0.0001**
Enthusiastic	14.0 (a)	15.4 (a)	17.8 (a)	16.8 (a)	15.9 (a)	0.791
Feminine	17.8 (b)	14.0 (b)	15.0 (b)	13.1 (b)	2.8 (a)	**<0.0001**
Happy	22.9 (a)	20.1 (a)	26.2 (a)	28.0 (a)	29.0 (a)	0.086
Inspiring	15.4 (a)	15.4 (a)	19.6 (ab)	20.6 (ab)	26.6 (b)	**0.004**
Nervous	7.5 (a)	9.8 (a)	7.9 (a)	7.0 (a)	4.2 (a)	0.152
Passive	8.4 (a)	7.9 (a)	8.4 (a)	8.9 (a)	7.9 (a)	0.993
Powerful	11.7 (ab)	10.7 (a)	8.4 (a)	5.1 (a)	18.7 (b)	**<0.0001**
Pretentious	14.0 (a)	10.3 (a)	9.8 (a)	11.7 (a)	7.5 (a)	0.135
Sophisticated	25.2 (b)	22.0 (ab)	29.9 (b)	23.8 (ab)	14.0 (a)	**0.0003**
Tense	6.1 (a)	3.7 (a)	7.0 (a)	3.7 (a)	7.9 (a)	0.108
Uninspired	12.1 (a)	13.6 (a)	9.3 (a)	10.3 (a)	8.9 (a)	0.368
Unique	16.4 (a)	16.4 (a)	17.3 (a)	26.2 (ab)	31.3 (b)	**<0.0001**
Youthful	8.4 (a)	7.0 (a)	9.3 (a)	10.7 (ab)	17.3 (b)	**0.0002**

S12C. CLUSTER 4.

**Term**	**PB Cheese**	**PB Fish**	**PB Meat**	**PB Meat 33%**	**PB Milk**	**p-value**
Adventurous	16.6 (a)	21.6 (a)	19.6 (a)	22.6 (a)	18.2 (a)	0.236
Boring	5.4 (a)	7.8 (a)	8.8 (a)	5.7 (a)	7.4 (a)	0.321
Classy	13.2 (a)	8.8 (a)	8.1 (a)	8.4 (a)	13.2 (a)	**0.025**
Comforting	15.9 (bc)	8.1 (a)	8.8 (ab)	11.1 (ab)	23.0 (c)	**<0.0001**
Dissatisfied	6.8 (a)	12.8 (b)	7.8 (ab)	8.1 (ab)	8.4 (ab)	**0.023**
Easygoing	15.9 (ab)	13.9 (ab)	13.2 (ab)	10.8 (a)	18.9 (b)	**0.022**
Energetic	4.4 (a)	5.4 (a)	4.7 (a)	2.4 (a)	18.2 (b)	**<0.0001**
Enthusiastic	23.3 (a)	18.2 (a)	19.6 (a)	19.6 (a)	16.9 (a)	0.255
Feminine	18.9 (b)	16.2 (b)	15.2 (b)	14.9 (b)	6.4 (a)	**<0.0001**
Happy	28.7 (a)	21.3 (a)	27.7 (a)	22.6 (a)	26.0 (a)	0.073
Inspiring	19.3 (a)	18.2 (a)	15.5 (a)	15.5 (a)	23.3 (a)	**0.034**
Nervous	5.4 (ab)	10.5 (b)	10.1 (ab)	9.1 (ab)	4.7 (a)	**0.003**
Passive	7.8 (a)	8.8 (a)	9.8 (a)	9.8 (a)	8.4 (a)	0.858
Powerful	8.1 (a)	9.5 (a)	9.1 (a)	8.4 (a)	18.2 (b)	**<0.0001**
Pretentious	11.5 (ab)	15.2 (b)	11.1 (ab)	10.1 (ab)	8.1 (a)	**0.045**
Sophisticated	29.4 (b)	30.4 (b)	31.4 (b)	31.1 (b)	12.5 (a)	**<0.0001**
Tense	3.4 (a)	7.1 (ab)	6.4 (ab)	8.4 (b)	5.1 (ab)	0.051
Uninspired	6.4 (a)	11.1 (a)	8.8 (a)	8.8 (a)	6.8 (a)	0.136
Unique	21.3 (ab)	17.6 (ab)	17.2 (a)	16.6 (a)	25.7 (b)	**0.010**
Youthful	15.9 (bc)	10.5 (ab)	12.8 (abc)	7.8 (a)	17.2 (c)	**0.0001**

S12D. CLUSTER 5.

Term	PB Cheese	PB Fish	PB Meat	PB Meat 33%	PB Milk	p-value
Adventurous	14.0 (a)	11.3 (a)	17.3 (a)	14.0 (a)	14.0 (a)	0.593
Boring	15.3 (b)	12.7 (ab)	12.0 (ab)	9.3 (ab)	4.7 (a)	**0.012**
Classy	8.0 (a)	7.3 (a)	8.7 (a)	6.0 (a)	6.7 (a)	0.864
Comforting	8.7 (a)	6.0 (a)	6.0 (a)	8.7 (a)	22.0 (b)	**<0.0001**
Dissatisfied	16.7 (b)	22.7 (b)	18.0 (b)	14.7 (ab)	6.0 (a)	**0.0001**
Easygoing	9.3 (a)	10.7 (a)	11.3 (a)	8.7 (a)	23.3 (b)	**<0.0001**
Energetic	2.7 (a)	2.7 (a)	4.7 (a)	6.0 (a)	13.3 (b)	**<0.0001**
Enthusiastic	16.7 (a)	10.7 (a)	14.0 (a)	13.3 (a)	18.0 (a)	0.309
Feminine	13.3 (b)	9.3 (ab)	10.7 (b)	12.0 (b)	2.7 (a)	**0.001**
Happy	15.3 (a)	14.0 (a)	19.3 (ab)	16.0 (ab)	26.7 (b)	**0.009**
Inspiring	9.3 (a)	8.0 (a)	11.3 (a)	8.7 (a)	23.3 (b)	**<0.0001**
Nervous	11.3 (ab)	13.3 (b)	8.0 (ab)	9.3 (ab)	3.3 (a)	**0.008**
Passive	5.3 (a)	6.7 (a)	5.3 (a)	12.0 (a)	5.3 (a)	0.065
Powerful	5.3 (a)	9.3 (ab)	8.7 (ab)	4.7 (a)	16.7 (b)	**0.001**
Pretentious	10.0 (ab)	11.3 (b)	8.7 (ab)	9.3 (ab)	3.3 (a)	**0.041**
Sophisticated	23.3 (b)	26.0 (b)	20.7 (ab)	24.0 (b)	10.0 (a)	**0.0003**
Tense	2.7 (a)	4.7 (a)	8.0 (a)	5.3 (a)	3.3 (a)	0.146
Uninspired	15.3 (ab)	21.3 (b)	19.3 (b)	14.0 (ab)	6.7 (a)	**0.0002**
Unique	16.0 (ab)	10.7 (a)	10.7 (a)	12.0 (a)	24.7 (b)	**0.0002**
Youthful	8.7 (a)	9.3 (a)	9.3 (a)	6.0 (a)	11.3 (a)	0.307

S12E. CLUSTER 6.

Term	PB Cheese	PB Fish	PB Meat	PB Meat 33%	PB Milk	p-value
Adventurous	27.3 (a)	20.3 (a)	23.1 (a)	24.5 (a)	18.9 (a)	0.314
Boring	4.9 (a)	6.3 (a)	7.7 (a)	8.4 (a)	5.6 (a)	0.619
Classy	15.4 (b)	10.5 (ab)	8.4 (ab)	5.6 (a)	10.5 (ab)	0.054
Comforting	14.7 (a)	7.7 (a)	18.2 (ab)	8.4 (a)	27.3 (b)	**<0.0001**
Dissatisfied	7.7 (ab)	15.4 (b)	9.8 (ab)	13.3 (b)	2.8 (a)	**0.001**
Easygoing	16.1 (ab)	13.3 (ab)	11.2 (ab)	9.8 (a)	21.0 (b)	**0.016**
Energetic	4.9 (a)	4.9 (a)	8.4 (a)	4.9 (a)	23.1 (b)	**<0.0001**
Enthusiastic	23.8 (b)	18.2 (ab)	19.6 (ab)	9.1 (a)	22.4 (b)	**0.008**
Feminine	14.7 (b)	9.8 (ab)	17.5 (b)	9.8 (ab)	4.9 (a)	**0.002**
Happy	28.0 (b)	18.2 (ab)	30.1 (b)	15.4 (a)	27.3 (ab)	**0.001**
Inspiring	22.4 (ab)	13.3 (a)	20.3 (ab)	14.7 (a)	26.6 (b)	**0.008**
Nervous	8.4 (ab)	10.5 (b)	7.7 (ab)	11.2 (b)	1.4 (a)	**0.006**
Passive	7.0 (a)	4.9 (a)	7.7 (a)	9.1 (a)	12.6 (a)	0.100
Powerful	7.0 (a)	11.2 (ab)	16.1 (ab)	11.2 (ab)	21.7 (b)	**0.001**
Pretentious	11.2 (a)	10.5 (a)	6.3 (a)	9.1 (a)	5.6 (a)	0.233
Sophisticated	28.0 (b)	23.1 (b)	21.7 (ab)	19.6 (ab)	11.2 (a)	**0.002**
Tense	2.1 (a)	2.8 (a)	4.9 (a)	2.8 (a)	2.1 (a)	0.497
Uninspired	7.7 (a)	8.4 (a)	4.9 (a)	11.2 (a)	5.6 (a)	0.211
Unique	25.9 (a)	16.1 (a)	21.7 (a)	18.2 (a)	28.0 (a)	**0.030**
Youthful	14.7 (a)	9.1 (a)	11.2 (a)	9.1 (a)	13.3 (a)	0.329

S12F. CLUSTER 7.

**Term**	**PB Cheese**	**PB Fish**	**PB Meat**	**PB Meat 33%**	**PB Milk**	**p-value**
Adventurous	23.1 (ab)	21.4 (ab)	22.0 (ab)	28.0 (b)	16.5 (a)	0.054
Boring	6.6 (a)	11.0 (a)	11.0 (a)	8.8 (a)	11.0 (a)	0.393
Classy	13.2 (a)	7.7 (a)	8.8 (a)	6.6 (a)	8.2 (a)	0.142
Comforting	12.6 (a)	13.2 (a)	11.0 (a)	13.7 (a)	16.5 (a)	0.571
Dissatisfied	12.1 (ab)	17.0 (b)	14.8 (ab)	6.6 (a)	12.6 (ab)	0.008
Easygoing	12.6 (a)	10.4 (a)	13.7 (a)	12.1 (a)	15.4 (a)	0.575
Energetic	3.8 (a)	7.7 (ab)	6.0 (a)	4.4 (a)	13.7 (b)	**0.0003**
Enthusiastic	15.4 (a)	8.8 (a)	12.6 (a)	12.6 (a)	15.4 (a)	0.221
Feminine	16.5 (b)	6.6 (a)	10.4 (ab)	9.9 (ab)	3.8 (a)	**<0.0001**
Happy	20.9 (a)	17.0 (a)	18.1 (a)	23.6 (a)	18.1 (a)	0.366
Inspiring	15.4 (ab)	10.4 (a)	15.4 (ab)	23.6 (b)	19.8 (ab)	**0.002**
Nervous	6.6 (a)	14.8 (b)	12.6 (ab)	7.1 (a)	10.4 (ab)	**0.010**
Passive	10.4 (a)	7.7 (a)	8.2 (a)	4.9 (a)	7.1 (a)	0.247
Powerful	12.1 (a)	14.3 (a)	12.1 (a)	10.4 (a)	13.2 (a)	0.779
Pretentious	10.4 (a)	8.8 (a)	11.0 (a)	9.3 (a)	6.6 (a)	0.532
Sophisticated	22.5 (b)	26.4 (b)	24.7 (b)	25.3 (b)	11.5 (a)	**<0.0001**
Tense	5.5 (a)	5.5 (a)	8.8 (a)	3.3 (a)	3.8 (a)	0.084
Uninspired	13.2 (b)	15.4 (b)	14.8 (b)	4.4 (a)	14.3 (b)	**0.001**
Unique	20.3 (a)	18.7 (a)	17.0 (a)	23.1 (ab)	32.4 (b)	**0.001**
Youthful	8.8 (a)	6.6 (a)	12.6 (a)	7.7 (a)	8.2 (a)	0.155

PART S13. Citation frequency (%) for situational use CATA terms. Results from Cochran Q test (final column) and post-hoc comparisons (Sheskin) by cluster (Cluster 2 to Cluster 7) for the five plant-based products in the study.

S13A. CLUSTER 2.

Term	Cheese	Fish	Meat	Meat33	Milk	p-value
When I want something I like	26.8 (a)	22.0 (a)	23.6 (a)	20.5 (a)	23.6 (a)	0.686
When I feel like trying something new	45.7 (a)	48.8 (a)	48.8 (a)	49.6 (a)	40.2 (a)	0.374
To move my diet in a more sustainable direction	39.4 (a)	37.8 (a)	43.3 (a)	42.5 (a)	34.6 (a)	0.419
When I want something healthy	33.1 (a)	28.3 (a)	33.9 (a)	31.5 (a)	40.2 (a)	0.181
As part of meals that I post on social media	11.8 (a)	10.2 (a)	11.8 (a)	13.4 (a)	11.0 (a)	0.877
To set a good example to those around me	12.6 (a)	14.2 (ab)	21.3 (ab)	24.4 (b)	19.7 (ab)	**0.014**
As a regular part of my diet	19.7 (a)	13.4 (a)	18.1 (a)	22.0 (ab)	33.1 (b)	**0.0002**
As part of easy and convenient meals	22.8 (a)	21.3 (a)	18.9 (a)	23.6 (a)	24.4 (a)	0.716

S13B. CLUSTER 3.

Term	Cheese	Fish	Meat	Meat33	Milk	p-value
When I want something I like	26.2 (a)	22.4 (a)	21.0 (a)	25.2 (a)	22.0 (a)	0.511
When I feel like trying something new	44.4 (a)	44.9 (a)	43.5 (a)	46.3 (a)	35.0 (a)	0.053
To move my diet in a more sustainable direction	37.9 (a)	41.1 (a)	46.7 (a)	46.3 (a)	38.8 (a)	**0.032**
When I want something healthy	31.8 (a)	33.2 (a)	40.2 (a)	38.3 (a)	40.7 (a)	0.069
As part of meals that I post on social media	11.7 (a)	11.7 (a)	10.7 (a)	9.3 (a)	11.2 (a)	0.854
To set a good example to those around me	16.4 (a)	16.4 (a)	24.8 (a)	18.7 (a)	20.6 (a)	**0.029**
As a regular part of my diet	22.4 (a)	19.6 (a)	24.8 (a)	29.9 (ab)	37.9 (b)	**<0.0001**
As part of easy and convenient meals	21.5 (a)	17.3 (a)	21.0 (a)	22.9 (a)	24.3 (a)	0.320

S13C. CLUSTER 4.

Term	Cheese	Fish	Meat	Meat33	Milk	p-value
When I want something I like	29.7 (b)	18.2 (a)	21.6 (ab)	23.0 (ab)	25.7 (ab)	**0.002**
When I feel like trying something new	41.9 (a)	45.9 (a)	44.6 (a)	45.9 (a)	41.9 (a)	0.586
To move my diet in a more sustainable direction	41.6 (ab)	45.6 (b)	40.5 (ab)	36.8 (ab)	35.1 (a)	**0.010**
When I want something healthy	37.5 (a)	32.1 (a)	34.1 (a)	38.9 (a)	38.5 (a)	0.160
As part of meals that I post on social media	13.2 (a)	12.2 (a)	13.2 (a)	11.1 (a)	11.8 (a)	0.859
To set a good example to those around me	23.6 (a)	23.6 (a)	23.0 (a)	24.0 (a)	22.6 (a)	0.989
As a regular part of my diet	30.1 (bc)	18.6 (a)	23.0 (ab)	23.0 (ab)	34.8 (c)	**<0.0001**
As part of easy and convenient meals	25.7 (ab)	22.3 (a)	27.0 (ab)	20.9 (a)	30.7 (b)	**0.009**

S13D. CLUSTER 5.

Term	Cheese	Fish	Meat	Meat33	Milk	p-value
When I want something I like	23.3 (ab)	16.7 (a)	17.3 (a)	17.3 (a)	30.0 (b)	**0.001**
When I feel like trying something new	44.7 (ab)	48.0 (b)	47.3 (b)	45.3 (b)	31.3 (a)	**0.002**
To move my diet in a more sustainable direction	30.7 (a)	36.0 (a)	41.3 (a)	38.0 (a)	31.3 (a)	0.059
When I want something healthy	36.0 (ab)	24.0 (a)	32.7 (ab)	35.3 (ab)	39.3 (b)	**0.014**
As part of meals that I post on social media	9.3 (a)	12.7 (a)	14.0 (a)	10.7 (a)	10.0 (a)	0.360
To set a good example to those around me	16.7 (a)	14.7 (a)	17.3 (a)	15.3 (a)	19.3 (a)	0.692
As a regular part of my diet	16.0 (a)	12.0 (a)	14.7 (a)	16.0 (a)	40.7 (b)	**<0.0001**
As part of easy and convenient meals	15.3 (a)	15.3 (a)	19.3 (a)	22.0 (a)	25.3 (a)	**0.024**

S13E. CLUSTER 6.

Term	Cheese	Fish	Meat	Meat33	Milk	p-value
When I want something I like	28.0 (bc)	21.7 (ab)	27.3 (bc)	13.3 (a)	34.3 (c)	**<0.0001**
When I feel like trying something new	53.1 (c)	47.6 (bc)	37.1 (ab)	48.3 (bc)	31.5 (a)	**0.0001**
To move my diet in a more sustainable direction	43.4 (a)	45.5 (a)	48.3 (a)	39.9 (a)	40.6 (a)	0.367
When I want something healthy	42.0 (ab)	32.2 (ab)	42.7 (b)	28.7 (a)	44.8 (b)	**0.002**
As part of meals that I post on social media	14.0 (a)	17.5 (a)	16.8 (a)	13.3 (a)	10.5 (a)	0.254
To set a good example to those around me	24.5 (a)	24.5 (a)	29.4 (a)	18.9 (a)	26.6 (a)	0.159
As a regular part of my diet	30.8 (ab)	23.8 (ab)	36.4 (b)	17.5 (a)	53.1 (c)	**<0.0001**
As part of easy and convenient meals	30.8 (a)	21.0 (a)	27.3 (a)	26.6 (a)	29.4 (a)	0.187

S13F. CLUSTER 7.

Term	Cheese	Fish	Meat	Meat33	Milk	p-value
When I want something I like	25.8 (a)	17.6 (a)	18.1 (a)	19.8 (a)	19.8 (a)	0.158
When I feel like trying something new	44.5 (a)	47.8 (a)	45.6 (a)	42.9 (a)	39.6 (a)	0.390
To move my diet in a more sustainable direction	34.6 (a)	33.5 (a)	41.2 (a)	42.3 (a)	43.4 (a)	**0.026**
When I want something healthy	34.6 (a)	25.8 (a)	36.3 (a)	31.9 (a)	31.9 (a)	0.113
As part of meals that I post on social media	9.9 (a)	12.1 (a)	10.4 (a)	13.2 (a)	8.8 (a)	0.422
To set a good example to those around me	23.1 (a)	18.7 (a)	19.8 (a)	25.3 (a)	25.8 (a)	0.153
As a regular part of my diet	22.0 (a)	18.1 (a)	24.2 (a)	23.6 (a)	28.6 (a)	0.087
As part of easy and convenient meals	25.3 (a)	18.7 (a)	22.5 (a)	24.7 (a)	23.1 (a)	0.446

References

1. Dunlap, R.E.; Van Liere, K.D.; Mertig, A.G.; Jones, R.E. New trends in measuring environmental attitudes: measuring endorsement of the new ecological paradigm: a revised NEP scale. *J. Soc. Issues* **2000**, *56*, 425-442.
2. Lounsbury, J.W.; Tornatzky, L.G. A scale for assessing attitudes toward environmental quality. *J. Soc. Psychol.* **1977**, *101*, 299-305.
3. Maloney, M.P.; Ward, M.P.; Braucht, G.N. A revised scale for the measurement of ecological attitudes and knowledge. *Am. Psychol.* **1975**, *30*, 787-790.
4. Goldammer, P.; Annen, H.; Stöckli, P.L.; Jonas, K. Careless responding in questionnaire measures: Detection, impact, and remedies. *Leadersh. Q.* **2020**, *31*, 101384.
5. Schonlau, M.; Toepoel, V. Straightlining in Web survey panels over time. *Surv. Res. Methods* **2015**, *9*, 125-137.
6. Pliner, P.; Hobden, K. Development of a scale to measure the trait of food neophobia in humans. *Appetite* **1992**, *19*, 105-120.
7. Cox, D.; Evans, G. Construction and validation of a psychometric scale to measure consumers' fears of novel food technologies: The food technology neophobia scale. *Food Qual. Prefer.* **2008**, *19*, 704-710.
